# Autophagy‐Targeting Fe–Cu Nanozyme for Tumor Immune Microenvironment Remodeling and Image‐Guided Cancer Immunotherapy

**DOI:** 10.1002/advs.202512575

**Published:** 2025-10-02

**Authors:** Li Yan, Chao Chen, Yu Liang, Xiaowan Huang, Jieying Qian, Hao Zhang, Li Zhang, Yingjia Li, Yunjiao Zhang

**Affiliations:** ^1^ Department of Medicine Ultrasonics Nanfang Hospital Southern Medical University Guangzhou 510515 P. R. China; ^2^ School of Medicine South China University of Technology Guangzhou 510006 P. R. China; ^3^ National Engineering Research Centre for Tissue Restoration and Reconstruction and Guangdong Provincial Key Laboratory of Biomedical Engineering South China University of Technology Guangzhou 510006 P. R. China

**Keywords:** autophagy inhibition, cancer immunotherapy, metal–organic frameworks, nanoenzyme, ultrasound imaging

## Abstract

The suppressive tumor immune microenvironment (TIME) is a critical driver of tumor progression, immune evasion, and therapy resistance. Despite the transformative potential of immunotherapy, autophagy within the TIME weakens immune surveillance by downregulating tumor cell surface major histocompatibility complex class I (MHC‐I) expression, thereby facilitating immune escape. Here, a novel nanozyme‐based strategy is reported to modulate autophagy and restore anti‐tumor immunity. Iron‐copper metal–organic frameworks (Fe‐Cu MOFs) are engineered with tunable peroxidase, glutathione peroxidase, and oxidase‐like activities, and an optimal Fe:Cu ratio that confers potent redox activity alongside robust inhibition of autophagic flux is identified. These MOF nanozymes selectively impair autophagy and restore MHC‐I expression in tumor cells, enhancing immune recognition. To further potentiate autophagic blockade, a multifunctional nanoplatform (FCMP@CQ/PFH) is developed by co‐loading low‐dose chloroquine (CQ) and encapsulating perfluorohexane (PFH) into the Fe–Cu MOFs. This combinatorial system couples nanozyme‐driven redox stress with lysosomal inhibition to synergistically suppress autophagy and reinvigorate anti‐tumor immune responses. Moreover, PFH facilitates ultrasound‐based real‐time visualization of therapeutic efficacy. Both in vitro and in vivo studies show that FCMP@CQ/PFH enhances cancer immunotherapy and suppresses metastasis. This study establishes a dual‐functional approach that combines autophagy inhibition with immune microenvironment reprogramming to circumvent immune resistance and advance precision cancer immunotherapy.

## Graphical abstract

In this study, a multifunctional iron‐copper metal–organic framework nanozyme platform co‐delivering chloroquine and perfluorohexane is engineered to circumvent tumor immune evasion by disrupting autophagic flux and restoring major histocompatibility complex class I presentation. This dual‐action nanoplatform orchestrates synergistic autophagy blockade via redox perturbation and lysosomal dysfunction, thereby reprogramming the immunosuppressive microenvironment to potentiate immunotherapeutic efficacy and attenuate metastatic dissemination.

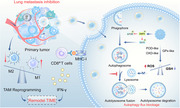

## 1 Introduction

Immunotherapy has revolutionized cancer treatment, yet tumor immune evasion and resistance remain formidable challenges driving disease progression.^[^ ^1^ ^]^ Central to this is the suppressive tumor immune microenvironment (TIME), which recruits pro‐tumor immune cells, depletes antigen‐specific cytotoxic T lymphocytes (CTLs), and promotes antigen loss or mutation, thereby undermining therapeutic efficacy.^[^ ^2^ ^]^ Targeting the TIME by modulating its cellular components has emerged as a promising strategy to restore immune function in cancer patients.^[^ ^3^ ^]^ Among these components, tumor‐associated macrophages (TAMs), the most abundant immune cells in the tumor microenvironment (TME), represent key targets for therapy.^[^ ^4^ ^]^ While M1‐polarized TAMs facilitate antitumor responses through antigen presentation and activation of tumor‐infiltrating lymphocytes (TILs), M2‐polarized TAMs promote immunosuppression, angiogenesis, and metastasis.^[^ ^5^ ^]^ Critically, the reversible polarization of TAMs offers an opportunity to reprogram the TIME to enhance immunotherapy responses.^[^ ^6^ ^]^ However, modulation of TAMs alone proves insufficient for complete tumor eradication, highlighting the necessity for combinatorial therapeutic approaches to augment antitumor immunity.^[^ ^7^ ^]^

Autophagy, a conserved lysosome‐mediated degradation pathway, exerts a dual role in cancer by enabling survival under metabolic stress while also being targetable to induce cancer cell death.^[^ ^8^ ^]^ Elevated autophagy within the tumor microenvironment contributes to immune evasion by downregulating major histocompatibility complex class I (MHC‐I) expression, impairing the recognition of tumor cells by CD8^+^ CTLs, and modulating T cell responses.^[^ ^9^ ^]^ Recent studies have elucidated how autophagy orchestrates tumor immune evasion through MHC‐I regulation.^[^ ^10^ ^]^ Multiple factors, including progranulin, N‐myc downstream‐regulated gene 1, and cholesterol 25‐hydroxylase suppress MHC‐I expression and antigen presentation via autophagy induction. This autophagy‐mediated MHC‐I degradation mechanism has been further validated across various tumor types, with receptor‐interacting serine/threonine‐protein kinase 2 and microtubule‐associated protein 1 light chain 3 (LC3) serving as additional regulatory nodes.^[^ ^11^ ^]^ These findings underscore autophagy modulation as a compelling therapeutic strategy to restore antitumor immunity. As such, autophagy inhibitors are gaining traction as potential immunotherapeutic adjuncts.^[^ ^12^ ^]^ Among these inhibitors, chloroquine (CQ), a clinically approved antimalarial agent, blocks autophagic flux by preventing autophagosome‐lysosome fusion and modulates TAM polarization.^[^ ^13^ ^]^ However, its effectiveness is hampered by poor bioavailability in acidic tumor environments and systemic toxicity, necessitating more precise and biocompatible delivery systems or alternative mechanisms to regulate autophagy within the TME.^[^ ^14^ ^]^

Recent advances in nanozyme technology offer a promising approach to address these challenges. Nanozymes, engineered nanomaterials that mimic natural enzymatic functions, can generate reactive oxygen species (ROS), deplete glutathione (GSH), and regulate redox homeostasis, thereby disrupting cellular pathways such as autophagy.^[^ ^15^ ^]^ In particular, metal‐organic frameworks (MOFs) have demonstrated peroxidase (POD)‐, oxidase (OXD)‐, and glutathione peroxidase (GPx)‐like activities, enabling them to simultaneously generate oxidative stress and interfere with autophagic flux.^[^ ^16^ ^]^ Among these materials, bimetallic MOFs exhibit enhanced catalytic performance and strong synergistic effects by integrating multiple metal centers within their frameworks.^[^ ^17^ ^]^ Notably, Fe–Cu bimetallic MOFs are effective nanoconstructs for the targeted delivery of ferroptosis inducers to tumor cells.^[^ ^18^ ^]^ For instance, a bimetallic Fe–Cu MOF in a responsive system demonstrated amplified ferroptosis by disrupting cellular iron and redox homeostasis for enhanced tumor therapy.^[^ ^19^ ^]^ Furthermore, the redox‐modulating properties of these MOF‐based nanozymes extend beyond direct cytotoxicity, enabling reprogramming of the tumor immune microenvironment through autophagy inhibition and subsequent immune activation, thus offering a multifaceted therapeutic strategy.^[^ ^20^ ^]^

**Scheme 1:**
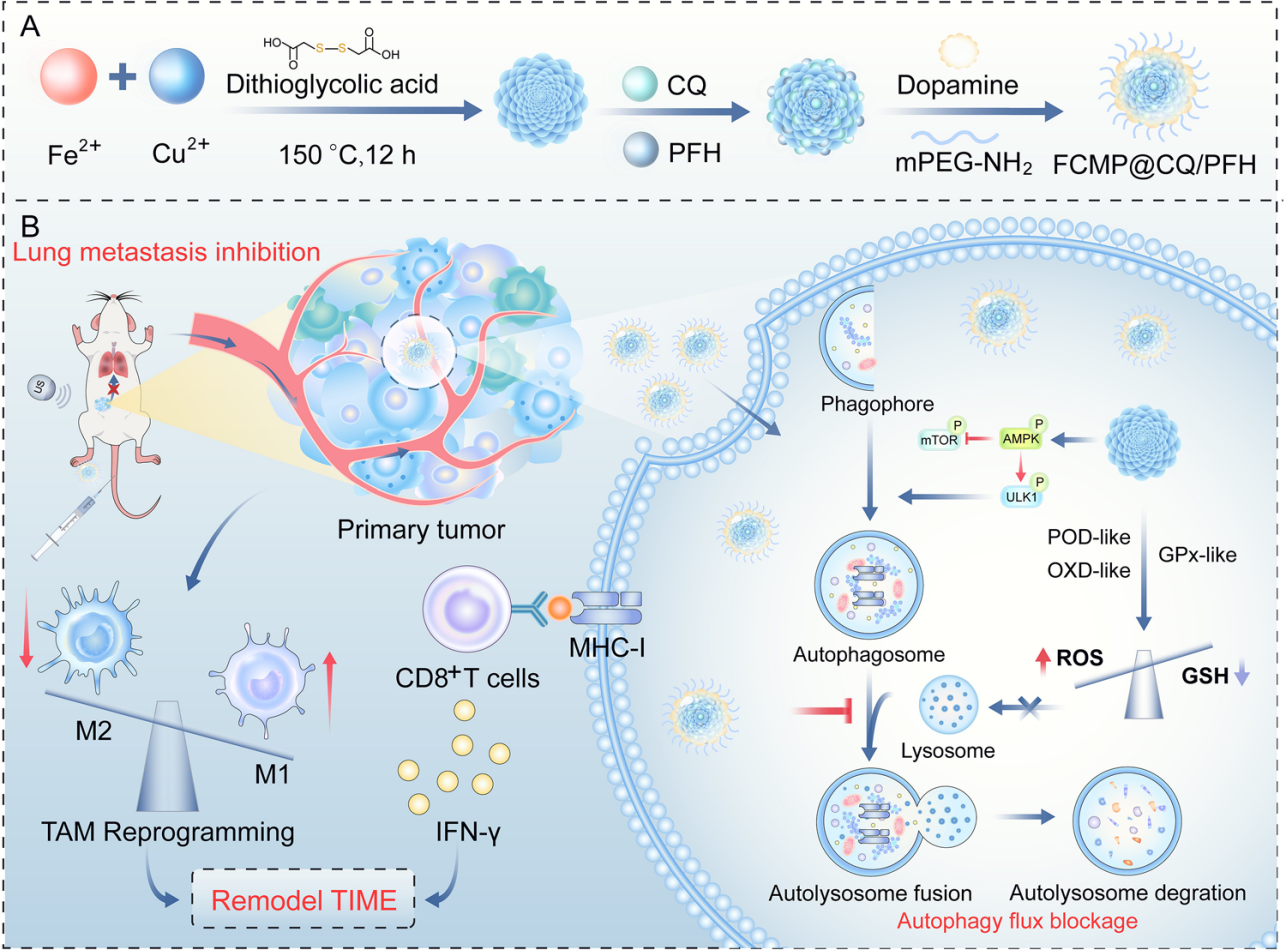
Ultrasound‐assisted FCMP@CQ/PFH nanoplatform with MOF‐based nanozyme activity for autophagy disruption and tumor immune microenvironment remodeling. A) Synthesis of FCMP@CQ/PFH. B) FCMP@CQ/PFH preferentially accumulates at the tumor site, where the MOF core generates ROS and depletes intracellular GSH via its intrinsic nanozyme‐like activity, inducing oxidative stress and disrupting autophagic flux by impairing autolysosome degradation. CQ further amplifies autophagy inhibition. These combined effects reprogram TAMs toward the M1 phenotype and enhance CD8⁺ T cell activation through upregulated MHC‐I expression and increased IFN‐γ secretion, thereby remodeling the tumor immune microenvironment and effectively suppressing tumor growth and metastasis.

In this study, we report the development of a multifunctional nanoparticle system based on iron‐ and copper‐containing MOFs (Fe‐Cu MOFs, FCM) encapsulating CQ and the imaging agent perfluorohexane (PFH), termed FCMP@CQ/PFH, as a synergistic autophagy inhibitor and immune modulator (**Scheme** **1**). This nanosystem leverages ultrasound (US)‐induced drug release to achieve targeted delivery, enabling spatiotemporally controlled autophagic flux inhibition and immunomodulation. Notably, FCM, endowed with intrinsic multienzyme‐mimetic activities, including POD‐, OXD‐, and GPx‐like functions, induces oxidative stress and redox imbalance, thereby promoting autophagy inhibition. Through this mechanism, FCM reprograms TAMs from the pro‐tumor M2 phenotype to the antitumor M1 phenotype and restores MHC‐I expression, thereby collectively boosting T cell‐mediated antitumor responses. The integration of PFH enables real‐time imaging via US‐guided therapy, offering dual therapeutic and diagnostic capabilities. In vitro and in vivo studies, including subcutaneous tumor and orthotopic breast cancer models, demonstrated that FCMP@CQ/PFH nanoparticles (NPs) under US achieved significant autophagic flux blockage, tumor suppression, reduced metastasis, and facilitated visualized treatment monitoring. Collectively, this work presents a redox‐enzyme‐integrated nanotherapeutic strategy that couples targeted autophagy inhibition with immune remodeling to augment the efficacy of cancer immunotherapy.

## 2 Results and Discussion

### 2.1 Fe‐Cu MOFs (11:4) Optimally Inhibit Autophagy and Enhance MHC‐I Expression

**Figure 1:**
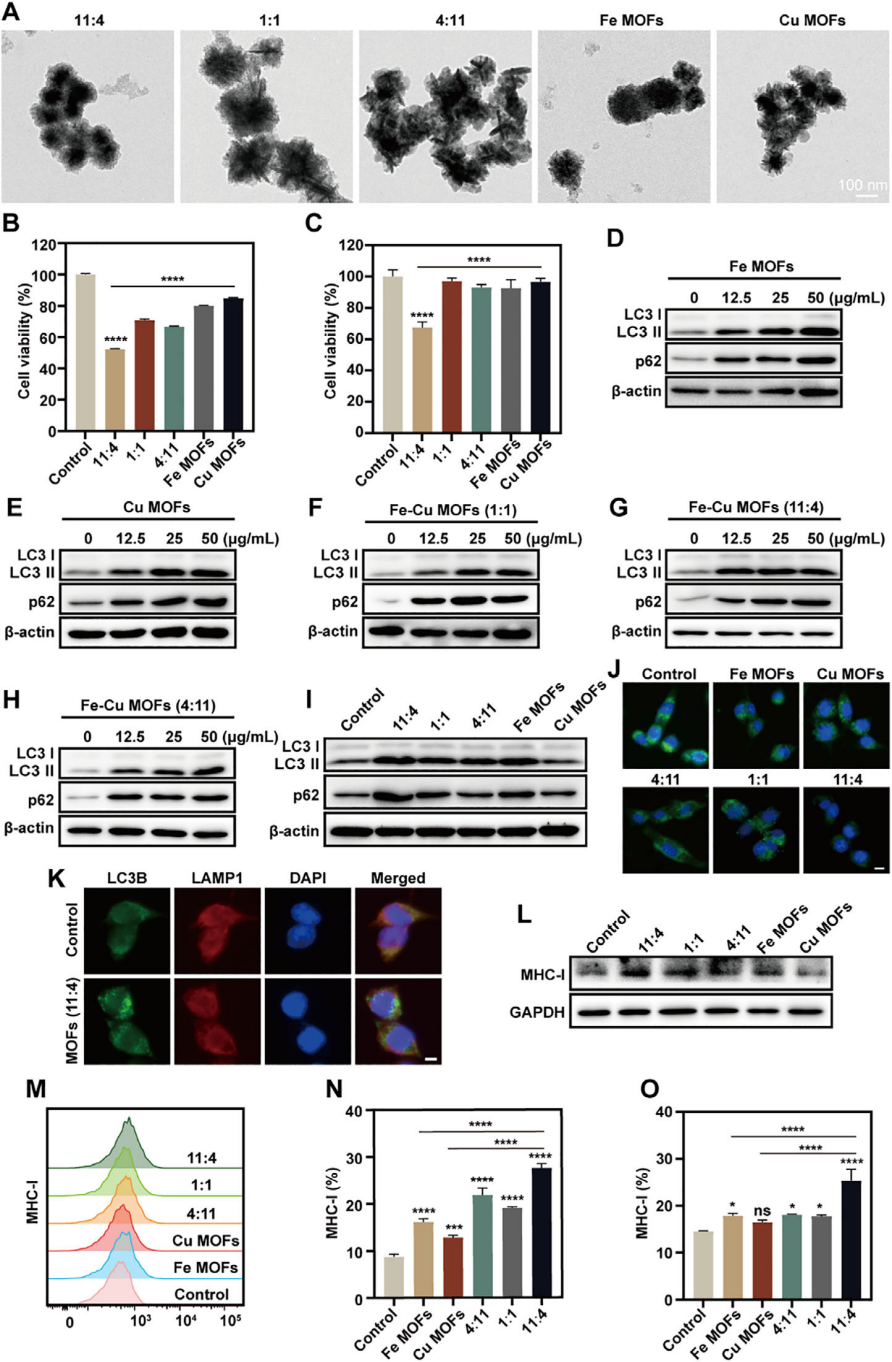
Autophagy‐related mechanisms induced by MOFs with different molar ratios of Fe and Cu. A) TEM images of MOFs with different molar ratios of Fe and Cu. Scale bar: 100 nm. B) Cell viability assessment treated with different MOFs formulations (11:4, 1:1, 4:11, Fe MOFs, Cu MOFs) at 50 µg mL^−1^ for 24 h in MC38 cells (B) and 4T1 cells (C). D–H) Western blot analysis of LC3 and p62 expression in MC38 cells treated with increasing concentrations of Fe MOFs (D), Cu MOFs (E), Fe–Cu MOFs (1:1) (F), Fe–Cu MOFs (11:4) (G), and Fe–Cu MOFs (4:11) (H), respectively, for 24 h. I) Western blot comparison of LC3 and p62 expression across all formulations at 25 µg mL^−1^ in MC38 cells for 24 h. J) Fluorescence images of Lyso‐Sensor Green DND‐189 in MC38 cells in various groups. Scale bar:10 µm. K) The immunofluorescence staining exhibited the colocalization and expression of LC3B and LAMP1 in the cytoplasm in MC38 cells treated with MOFs (11:4) at 25 µg mL^−1^ for 24 h. Scale bar: 5 µm. L) Western blot analysis of MHC‐I expression in MC38 cells following treatment with various MOFs at 25 µg mL^−1^ for 24 h. M) Flow cytometry overlay histogram of surface MHC‐I expression under different treatments. N) Quantification of MHC‐I‐positive cell percentage in MC38 and O) 4T1 cells treated with various MOFs for 24 h. All data are expressed as mean ± SD (n = 3). Statistical significance was determined by one‐way ANOVA with Tukey's test. ^*^ *p* < 0.05, ^**^ *p* < 0.01, ^***^ *p* < 0.001, ^****^ *p* < 0.0001.

To rationally identify the optimal Fe–Cu molar ratio for disrupting autophagic flux and enhancing immune activation, a series of Fe–Cu MOFs were synthesized at various molar ratios (Fe:Cu = 11:4, 1:1, and 4:11), along with monometallic Fe MOFs and Cu MOFs. As shown in **Figure** **1**A, transmission electron microscopy (TEM) images reveal the morphologies of these MOFs. To evaluate their biological activity, cell viability assays were performed, which demonstrated that the Fe–Cu MOFs with a molar ratio of 11:4 exhibited the most significant cytotoxicity against MC38 and 4T1 cells among all tested formulations (Figure 1B,C). To elucidate the underlying mechanism of cytotoxicity, we examined autophagy markers by Western blot analysis. Elevated LC3‐II levels may indicate either enhanced autophagosome formation or impaired autophagosomal maturation and degradation.^[^ ^21^ ^]^ Sequestosome‐1 (p62) binds directly to LC3 and is selectively recruited into autophagosomes, where it is efficiently degraded under normal autophagic flux; however, its levels accumulate when autophagy is impaired.^[^ ^22^ ^]^ Treatment with Fe MOFs, Cu MOFs, and Fe–Cu MOFs at various ratios led to a dose‐dependent accumulation of both LC3‐II and p62 (Figure 1D–H; Figure S1, Supporting Information), indicating that these MOFs effectively block autophagic flux. Notably, comparative assessment showed that Fe–Cu MOFs (11:4) induced the highest degree of p62 accumulation among all MOFs tested (Figure 1I; Figure S2, Supporting Information), reflecting its superior ability to impair autophagic flux. To further investigate the mechanism underlying the autophagic flux blockade, we examined lysosomal function, as intra‐lysosomal pH is a critical determinant of lysosomal activity. We performed Lyso‐Sensor Green staining, which exhibits decreased fluorescence intensity upon lysosomal alkalinization. As shown in Figure 1J, treatment with Fe–Cu MOFs at an 11:4 ratio resulted in the most pronounced reduction in Lyso‐Sensor Green fluorescence compared to control and other treatment groups, indicating significant lysosomal alkalinization. To further examine the effect of Fe–Cu MOFs (11:4) on autophagic flux, we next examined the autophagosome‐lysosome fusion process by tracking the late endosome/lysosome marker lysosomal‐associatedmembrane protein 1 (LAMP1) to detect its colocalization with the autophagosomal marker LC3. As shown in Figure 1K, treatment with Fe–Cu MOFs (11:4) led to a notable accumulation of LC3 puncta in MC38 cells; however, compared to the control group, the majority of these LC3 puncta (green) showed limited colocalization with the lysosomal marker LAMP1 (red). These findings suggest that Fe–Cu MOFs (11:4) block the fusion between autophagosomes and lysosomes, thereby impairing autophagic flux. Given that autophagy dysfunction is associated with enhanced MHC‐I expression,^[^ ^23^ ^]^ we next assessed MHC‐I surface expression via flow cytometry and western blotting. Western blot analysis (Figure 1L) revealed that treatment with MOFs, particularly the 11:4 Fe–Cu ratio formulation, significantly upregulated total MHC‐I protein expression in MC38 cells compared to the control and other MOF groups. Consistent with this, flow cytometric analysis (Figure 1M) demonstrated a marked increase in MHC‐I surface expression following Fe–Cu MOFs (11:4) treatment. Quantitative analysis of flow cytometry data in both MC38 and 4T1 cells (Figure 1N,O) confirmed that Fe–Cu MOFs with the 11:4 ratio induced the most pronounced elevation in surface MHC‐I levels among all tested formulations. Collectively, these results suggest that Fe–Cu MOFs (11:4) not only impair autophagic flux but also enhance MHC‐I expression, thereby potentially promoting immunogenic remodeling of the tumor microenvironment.

### 2.2 Characterization and Enzymatic Activity Evaluation of FCMP

**Figure 2:**
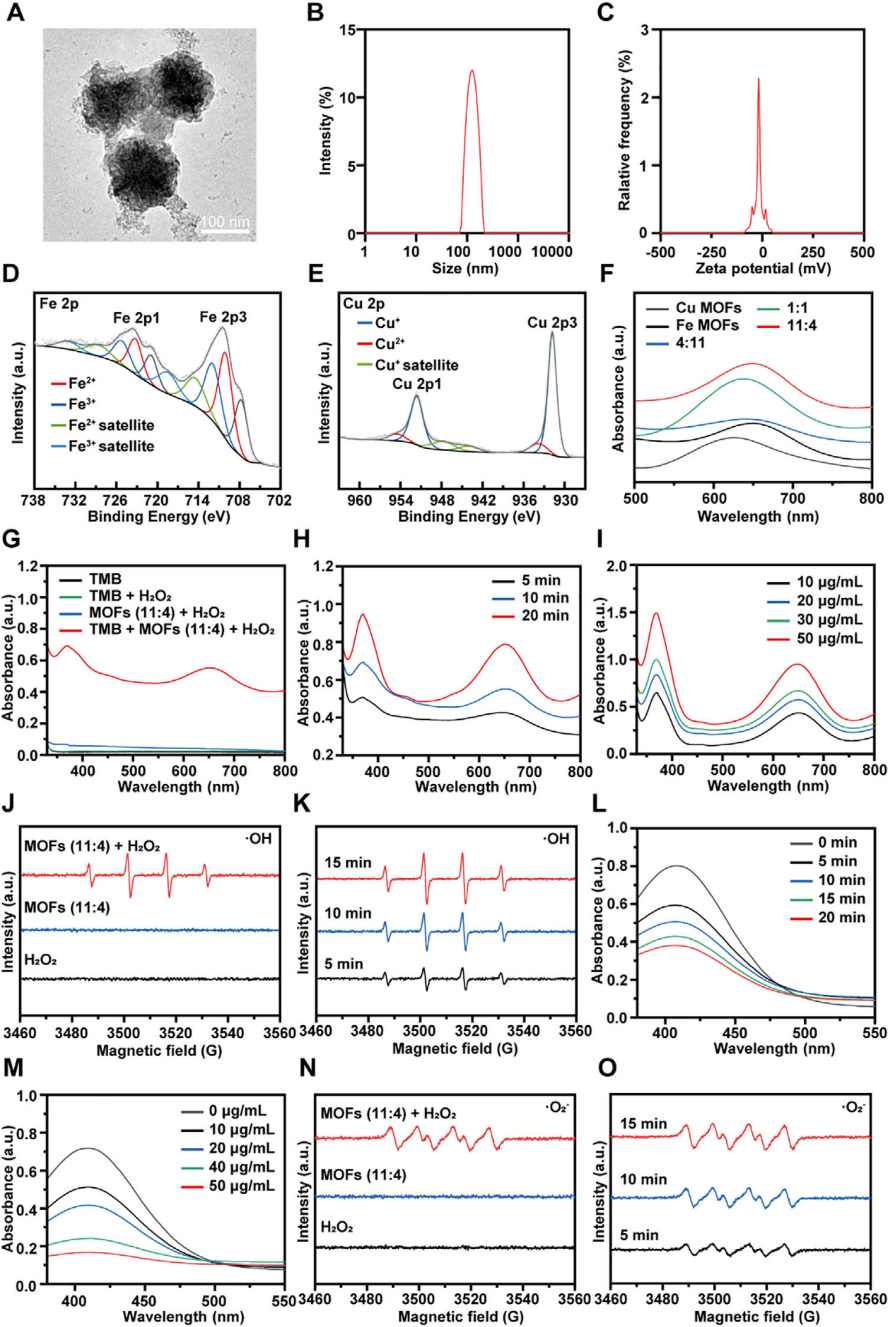
Characterization and catalytic activities of FCMP nanozymes. A) TEM image of FCMP NPs. Scale bar: 100 nm. B) DLS analysis showing the hydrodynamic diameter distribution of FCMP NPs. C) Zeta potential measurement of FCMP NPs. D) High‐resolution XPS spectra of Fe2p. E) High‐resolution XPS spectra of Cu2p. F) UV–vis absorption spectra comparing the POD‐like activities of different MOFs compositions. G) UV–vis absorption spectra of TMB solution containing FCMP NPs in the presence of H_2_O_2_ (pH 6.5). H) Time‐dependent TMB oxidation catalyzed by FCMP NPs, monitored by absorbance changes. I) Concentration‐dependent UV–vis absorbance spectra of TMB solutions treated with FCMP NPs. J) DMPO/·OH generation detected by ESR spectra. K) Time‐dependent ESR spectra demonstrating the kinetics of ·OH generation. L) Time‐dependent UV–vis absorbance spectra of DTNB solutions treated with FCMP NPs. M) Concentration‐dependent UV–vis absorbance spectra of DTNB solutions treated with FCMP NPs. N) DMPO/·O_2_^−^ generation detected by ESR spectra. O) Time‐dependent ESR spectra demonstrating the kinetics of ·O_2_^−^ generation.

FCMP NPs, derived from Fe–Cu MOFs with an optimized 11:4 molar ratio—which our previous studies identified as the most effective formulation for autophagy disruption and MHC‐I upregulation—were synthesized using hydrothermal methods, then coated with polydopamine (PDA) and functionalized with mPEG‐NH_2_, resulting in a final particle size of approximately 130 nm as measured by TEM (**Figure** **2**A) and dynamic light scattering (DLS) (Figure 2B). Moreover, zeta potential measurements indicated a surface charge of −17.10 mV for FCMP (Figure 2C). Inductively coupled plasma optical emission spectrometry (ICP‐OES) analysis further confirmed that the Fe, Cu, and S contents were approximately 22.95%, 11.84%, and 2.88%, respectively. To further elucidate the elemental composition and oxidation states, high‐resolution XPS spectra of Cu and Fe were obtained. As shown in Figures 2D,E and S3 (Supporting Information), Cu existed as Cu^2^⁺ (933.88 eV) and Cu⁺ (931.8 eV), while Fe exhibited two valence states, Fe^2^⁺ (710.04 eV) and Fe^3^⁺ (711.9 eV). X‐ray diffraction (XRD) characterization confirmed the structure of the as‐prepared MOFs (11:4), revealing a diffraction pattern that exclusively matched CuFe_2_S_3_ with no impurity phases detected (Figure S4, Supporting Information). To understand the pyrolysis process, thermogravimetric‐derivative thermogravimetry (TG‐DTG) analysis of MOFs was performed (Figure S5, Supporting Information). The curves reveal three major weight loss stages: ≈5.1% (RT‐130 °C), associated with the removal of adsorbed water and residual solvents; ≈22.4% (130–620 °C), attributed to framework decomposition with a maximum rate at 367.6 °C; and ≈7.4% (620–800 °C), corresponding to further carbonization and the release of heteroatoms from the framework or potential phase transformation under an inert atmosphere. Importantly, transition metals with multivalent states, including Fe^2^⁺/Fe^3^⁺ and Cu⁺/Cu^2^⁺, possess enzyme‐mimetic catalytic properties that are promising for tumor‐specific therapy. In particular, the disruption of redox homeostasis within the TME can exert cytotoxic effects on tumor cells.^[^ ^24^ ^]^ To identify the optimal Fe–Cu ratio for catalytic activity, the POD‐like activities of various Fe–Cu MOFs were evaluated using the 3,3′,5,5′‐tetramethylbenzidine (TMB) colorimetric assay at pH 6.5. In the presence of H_2_O_2_, MOFs nanozymes could catalyze the oxidation of TMB to blue oxidized TMB (oxTMB), which exhibited an UV–vis absorption peak at 652 nm, indirectly indicating the generation of ·OH.^[^ ^25^ ^]^ Among the tested formulations, the MOFs with an Fe: Cu ratio of 11:4 exhibited the strongest catalytic activity, as evidenced by the highest absorbance at 652 nm (Figure 2F). These results indicate that the 11:4 Fe–Cu MOFs exhibit the most potent POD‐like activity, suggesting enhanced ·OH generation due to an optimized redox‐active composition. Building upon these findings, the POD‐like activity of FCMP (11:4) nanozymes was further examined under varying concentrations and reaction times. To further validate the enzymatic performance of FCMP, the POD‐like activity was systematically evaluated using the TMB‐H_2_O_2_ system. The catalytic activity showed clear dependence on both FCMP concentration and reaction time. As demonstrated in Figure 2G, FCMP exhibited significant catalytic activity in the presence of H_2_O_2_, with the TMB + MOFs (11:4) + H_2_O_2_ system showing substantial absorbance enhancement compared to individual components. Furthermore, as shown in Figure 2H, oxTMB absorbance increased over time. Concentration‐dependent studies further confirmed the dose‐responsive nature of FCMP's catalytic activity. Figure 2I shows that increasing FCMP concentrations from 10 to 50 µg mL^−1^ resulted in progressively enhanced absorbance peaks, indicating improved TMB oxidation efficiency. To further verify the ROS‐generating capabilities of the Fe–Cu MOFs, electron spin resonance (ESR) spectroscopy was performed using 5,5‐dimethyl‐1‐pyrroline N‐oxide (DMPO) as the spin‐trapping agent. As shown in Figure 2J, a characteristic quartet signal of DMPO‐·OH was observed only in the presence of both MOFs (11:4) and H_2_O_2_, indicating that MOFs (11:4) generated hydroxyl radicals. Moreover, time‐dependent ESR spectra (Figure 2K) further confirmed the progressive enhancement of ·OH signal intensity with prolonged reaction time, suggesting continuous ·OH production. To further evaluate the GPx‐like catalytic activity of FCMP nanoparticles, their GSH‐depleting capability was investigated. 5,5′‐Dithiobis (2‐nitrobenzoic acid) (DTNB) was employed as a chromogenic probe, which reacts with GSH to produce the yellow‐colored 5‐thio‐2‐nitrobenzoic acid (TNB), characterized by a distinct absorbance peak at 412 nm.^[^ ^26^ ^]^ As shown in Figure 2L, the absorbance at 412 nm gradually declined over time in the presence of FCMP NPs, indicating a time‐dependent depletion of GSH. Furthermore, a concentration‐dependent study was performed to evaluate the impact of varying FCMP NPs levels on GSH consumption. As presented in Figure 2M, increasing concentrations of FCMP NPs led to a progressive reduction in the absorbance at 412 nm, suggesting enhanced GSH depletion with higher NP concentrations. These findings collectively demonstrate the robust GPx‐like catalytic activity of FCMP NPs and their potential to disrupt intracellular redox homeostasis through sustained GSH consumption. As shown in Figure 2N, the OXD‐like activity of MOFs (11:4) induced the production of ·O_2_^−^, and ·O_2_^−^ generation was significantly enhanced with the addition of H_2_O_2_ because MOFs (11:4) nanozymes catalyze the decomposition of H_2_O_2_ to generate O_2_ during this process. Consistently, the time‐dependent ESR analysis (Figure 2O) demonstrated a marked increase in ·O_2_^−^ signal over time. Taken together, these results indicate that MOFs (11:4) exhibit multiple enzyme‐mimetic catalytic activities—POD‐like, GPx‐like, and OXD‐like—that can induce cytotoxic ROS generation and redox imbalance, thus highlighting their therapeutic potential in tumor microenvironment modulation.

### 2.3 FCMP Triggers Autophagy Initiation via AMPK‐mTOR Pathway and Blocks Late‐stage Autophagic Flux

**Figure 3:**
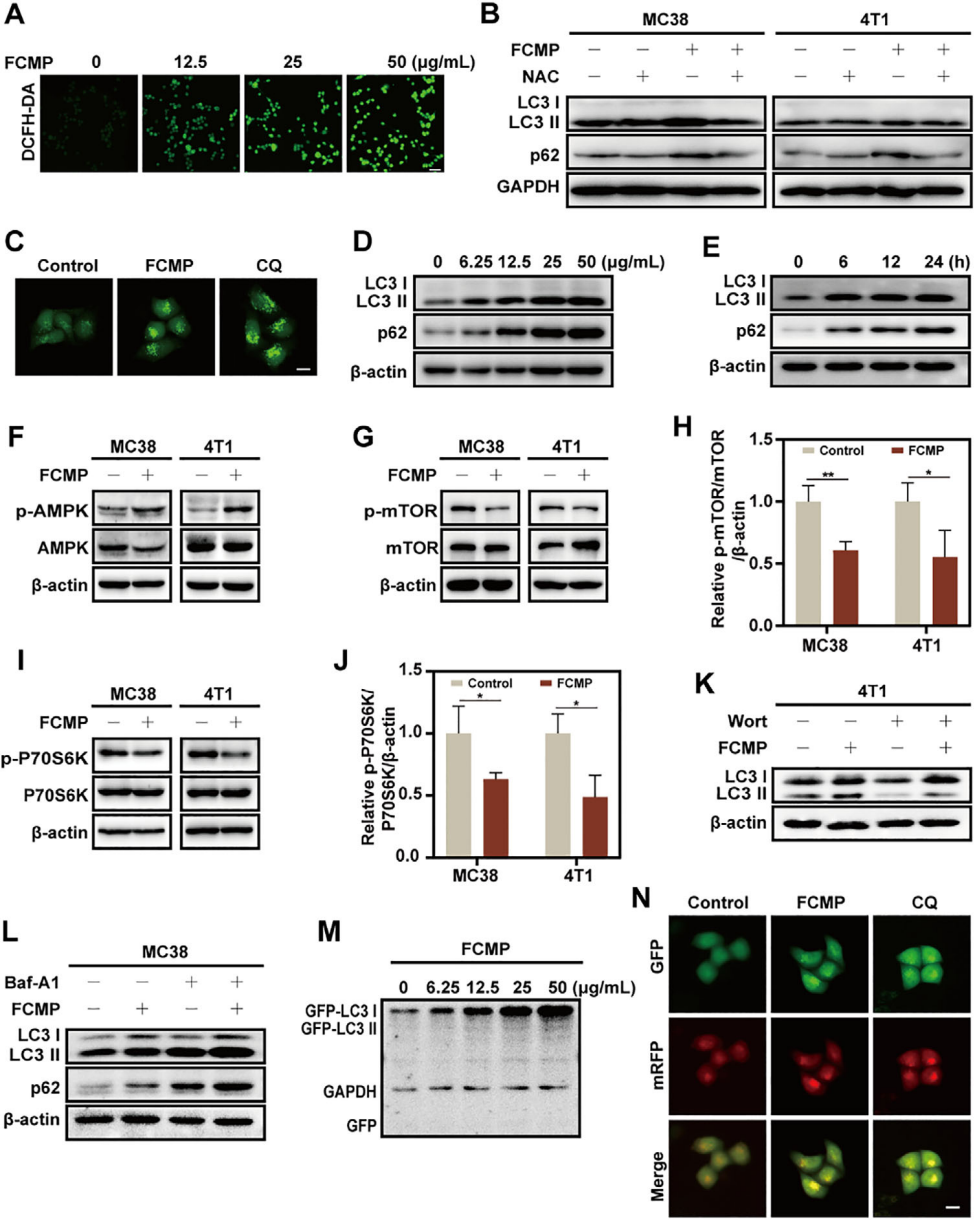
The mechanism of autophagic flux inhibition induced by FCMP in vitro. A) Intracellular ROS levels in MC38 cells treated with FCMP (11:4) at indicated concentrations (0, 12.5, 25, and 50 µg mL^−1^) for 24 h, as detected by DCFH‐DA staining. Scale bar: 10 µm. B) Western blotting of LC3 and p62 levels in MC38 and 4T1 cells after treatment with FCMP at 25 µg mL^−1^ and NAC at 2 mM for 24 h. C) GFP‐LC3 dot formation of HeLa‐LC3 cells with different treatments for 24 h (Scale bar: 20 µm). D) Western blotting analysis of LC3 and p62 proteins extracted from 4T1 cells treated with different doses of FCMP for 24 h. E) Western blotting of LC3 and p62 levels in MC38 cells treated with FCMP at 25 µg mL^−1^ for different times. F) MC38 and 4T1 cells treated with PBS (control) and 25 µg mL^−1^ FCMP for 24 h analyzed by AMPK and phospho‐AMPK western blotting. G) MC38 and 4T1 cells treated with PBS (control) and 25 µg mL^−1^ FCMP for 24 h were analyzed for mTOR signaling proteins expression by western blotting analysis for levels of total and phospho(p)‐mTOR and I) P70S6K. H), J) Quantified results for p‐mTOR/mTOR/β‐actin and p‐P70S6K/P70S6K/β‐actin ratios in Figure 3G,I. K) Western blotting of LC3 in 4T1 cells treated with 25 µg mL^−1^ FCMP for 24 h in the presence or absence of 1 mM wortmannin. L) MC38 cells were treated with 25 µg mL^−1^ FCMP for 24 h in the presence or absence of 400 nM bafilomycin A1 (added 4 h before cell harvest). M) Western blotting of GFP in HeLa GFP‐LC3 cells treated with different concentrations of FCMP for 24 h. N) Fluorescence imaging of GFP‐mRFP‐LC3 stably expressed in HeLa cells after different treatments for 24 h (Scale bar: 20 µm). Data are shown as mean ± SD (*n* = 3). Statistical significance was determined by unpaired two‐tailed Student's t‐test. ^*^p < 0.05, ^**^p < 0.01, ^***^p < 0.001, ^****^ *p* < 0.0001.

Having established the robust POD‐like, GPx‐like, and OXD‐like catalytic activities of FCMP (11:4) nanozymes, we next explored whether the generated oxidative stress was involved in the regulation of autophagy. Intracellular ROS levels were first measured using the DCFH‐DA probe. As shown in **Figure** **3**A, FCMP NPs induced a concentration‐dependent increase in fluorescence intensity, indicating elevated intracellular ROS levels. To determine whether oxidative stress contributes to FCMP‐induced impairment of autophagic flux, we co‐treated cells with the ROS scavenger N‐acetylcysteine (NAC) and FCMP, and examined the levels of LC3 and p62. As shown in Figure 3B, FCMP treatment led to an accumulation of both LC3‐II and p62 in MC38 cells, indicating a blockade of autophagosome‐lysosome fusion. Co‐treatment with NAC significantly reduced p62 levels and decreased LC3‐II accumulation, suggesting that scavenging ROS can restore autophagic flux. A similar trend was observed in 4T1 cells, where NAC reduced the FCMP‐induced increase in p62, although its effect on LC3‐II accumulation was less marked. These results suggest that ROS generation plays a critical role in FCMP‐mediated blockade of autophagic flux. Given that oxidative stress is known to regulate autophagy through multiple signaling pathways, particularly the AMPK‐mTOR axis.^[^ ^27^ ^]^ We next investigated whether FCMP‐induced ROS generation modulates autophagy by altering AMPK and mTOR phosphorylation status. To visualize autophagosome formation, we established a HeLa‐GFP‐LC3 cell line stably expressing enhanced green fluorescent protein‐tagged LC3 (GFP‐LC3). Under basal conditions, GFP‐LC3 is diffusely localized in the cytoplasm; however, during autophagy, it forms distinct green puncta indicative of autophagosome formation.^[^ ^28^ ^]^ Following FCMP treatment, GFP‐LC3 puncta were observed in HeLa‐GFP‐LC3 cells, resembling the effect induced by CQ and signifying autophagosome formation (Figure 3C). This was further substantiated by the conversion of LC3‐I to its lipid‐modified form, LC3‐II, and a dose‐dependent accumulation of p62 in 4T1 and MC38 cells through western blot. Both LC3‐II and p62 levels were significantly elevated following FCMP treatment in a dose‐dependent manner, with the most pronounced effect observed at 50 µg mL^−1^ (Figure 3D; Figure S6, Supporting Information). Time‐course experiments further confirmed the dose‐ and time‐dependent effects on autophagic markers in both cell lines (Figure 3E; Figure S7, Supporting Information). Collectively, these results indicate that FCMP triggers autophagy initiation. To elucidate the mechanism by which FCMP regulates autophagosome formation, we first examined the activation status of AMP‐activated protein kinase (AMPK), a central energy sensor and redox‐responsive kinase involved in autophagy regulation. FCMP treatment markedly increased AMPK phosphorylation at Thr172 in both MC38 and 4T1 cells, indicating its activation (Figure 3F). As a direct downstream target of AMPK, UNC‐51‐like kinase 1 (ULK1) was also activated, as evidenced by increased phosphorylation at Ser555, which is essential for autophagy initiation (Figure S8, Supporting Information). We next examined its effects on the mechanistic target of rapamycin (mTOR) signaling pathway, a central regulator of autophagy that inhibits autophagy initiation.^[^ ^29^ ^]^ FCMP suppressed phosphorylation of mTOR and its downstream substrate p70S6 kinase in MC38 and 4T1 cells, suggesting that its effect on autophagosome formation is mTOR‐dependent but independent of the AKT pathway (Figure 3G–J; Figure S9, Supporting Information). These findings suggest that FCMP induces autophagy initiation via ROS‐mediated activation of the AMPK–ULK1 axis, which subsequently inhibits mTOR signaling. To further explore the autophagic process, we employed autophagy inhibitors wortmannin (Wort), which blocks autophagosome formation, and bafilomycin A1 (Baf‐A1), which inhibits autophagosome‐lysosome fusion.^[^ ^30^ ^]^ Wortmannin significantly reduced FCMP‐induced autophagy initiation (Figure 3K; Figure S10, Supporting Information), while Baf‐A1 prevented LC3‐II degradation. Importantly, the presence of Baf‐A1 abrogated any further increase in LC3‐II levels upon FCMP treatment in both cell lines (Figure 3L; Figure S11, Supporting Information), suggesting that FCMP impairs autophagy at a late stage, akin to Baf‐A1. To investigate autophagic degradation, we utilized GFP‐LC3 to monitor autophagic flux by detecting free GFP. FCMP treatment increased GFP‐LC3 puncta in HeLa cells but failed to generate detectable free GFP, even after prolonged exposure (Figure 3M; Figure S12, Supporting Information). This indicates that FCMP‐mediated autophagy inhibition occurs at the late maturation stage, preventing autolysosome degradation. Next, we used the cell‐permeant Magic Red to assess the activity of cathepsin B, a lysosomal marker enzyme. FCMP‐treated MC38 cells exhibited a marked reduction in Magic Red fluorescence (Figure S13, Supporting Information), indicating impaired cathepsin B activity within lysosomes. Collectively, these results suggest that FCMP induces lysosomal dysfunction, thereby hindering autophagosome–lysosome fusion. Additionally, HeLa cells stably expressing GFP‐mRFP‐LC3, a dual‐fluorescent LC3 fusion protein, were used to simultaneously distinguish between autophagosomes (GFP^+^ mRFP^+^) and autolysosomes (mRFP^+^ only). In control cells, normal autophagic flux resulted in the formation of red‐only puncta due to the maturation of autolysosomes, where the acidic environment led to GFP degradation. In contrast, FCMP treatment resulted in a marked increase in yellow puncta (GFP^+^ mRFP^+^), signifying disrupted autophagosome‐lysosome fusion (Figure 3N). Taken together, these results demonstrate that FCMP initiates autophagy through ROS‐mediated activation of the AMPK‐ULK1 axis and inhibition of mTOR signaling while concurrently disrupting autophagosome maturation.

### 2.4 Synthesis and Characterization of FCMP@CQ/PFH and Its Imaging Performance

**Figure 4:**
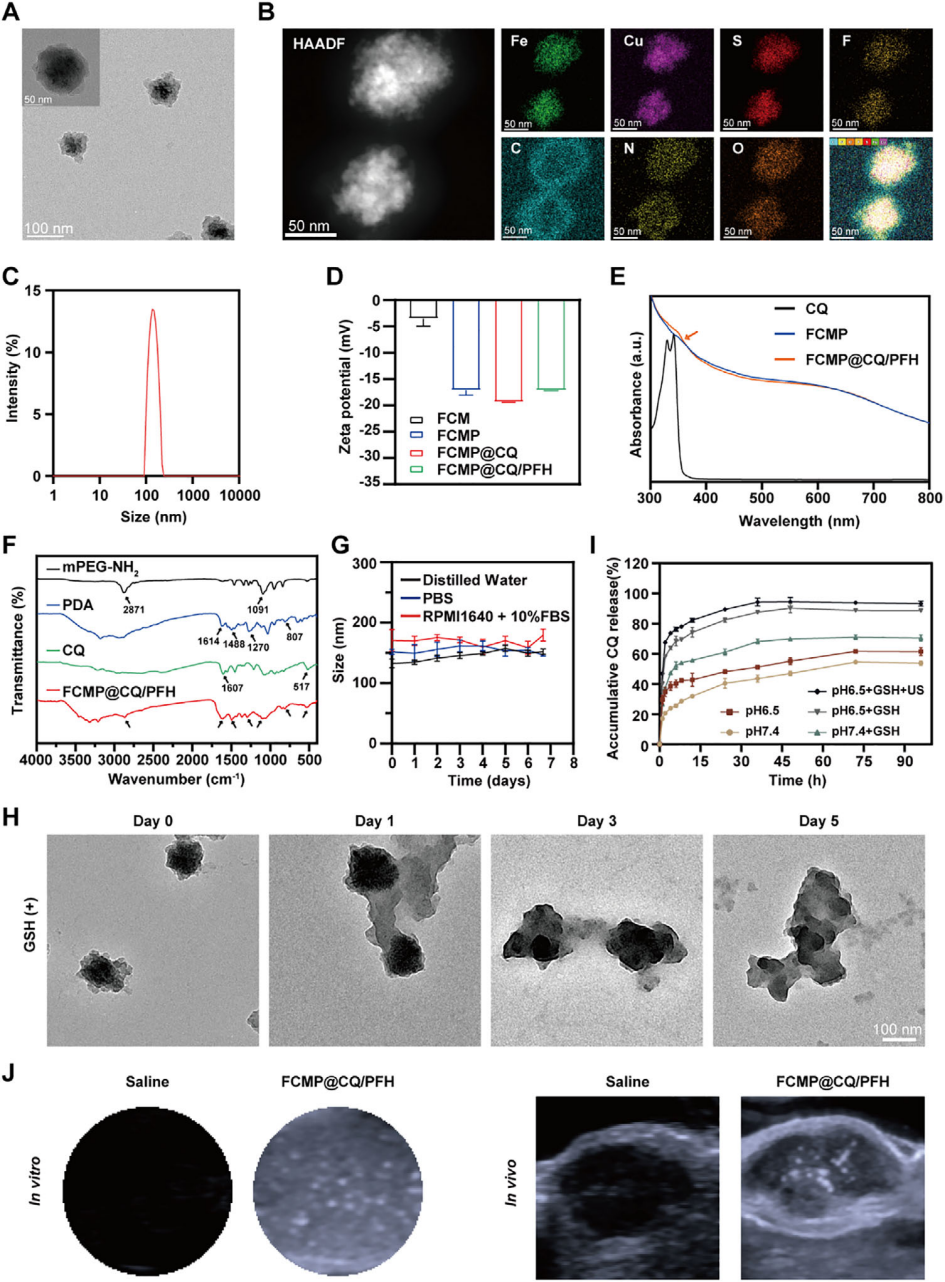
Characterization and ultrasound imaging efficacy of FCMP@CQ/PFH in vitro and in vivo. A) Representative TEM images of FCMP@CQ/PFH. B) EDS  elemental mapping of FCMP@CQ/PFH. C) DLS measurements of FCMP@CQ/PFH. D) Zeta potential of FCM, FCMP, FCMP@CQ, and FCMP@CQ/PFH. E) UV–vis absorption spectra of CQ, FCMP, and FCMP@CQ/PFH. F) FTIR spectra of FCMP@CQ/PFH. G) Stability of FCMP@CQ/PFH in different media (n = 3, mean ± SD). H) TEM images of biodegradable FCMP@CQ/PFH immersed in 10 mM GSH aqueous solution for days 0, 1, 3, and 5. I) In vitro release profiles of CQ from FCMP@CQ/PFH under different conditions (*n* = 3, mean ± SD). J) US imaging efficacy of FCMP@CQ/PFH in vitro and in vivo compared with saline.

CQ, a classic lysosomotropic agent, inhibits lysosomal degradation by increasing the lysosomal pH.^[^ ^31^ ^]^ However, long‐term CQ treatment has been associated with significant toxicity, including dermatological and nephrotoxic effects, as evidenced by clinical trials.^[^ ^32^ ^]^ In this study, we investigated the molecular mechanisms underlying autophagy inhibition mediated by FCMP, a novel nano‐autophagy flux inhibitor. The combination of FCMP with CQ holds promise as a potent antitumor therapeutic agent. Therefore, we synthesized FCMP@CQ/PFH. Initially, we synthesized FCM and subsequently loaded it with CQ and PFH through hydrophobic interactions and a negative pressure method. The nanoparticle surface was then modified with a PDA layer and mPEG‐NH_2_, resulting in FCMP@CQ/PFH. TEM analysis confirmed the spherical morphology of the nanoparticles, with the PDA layer clearly visible, indicating successful surface modification and enhanced stability by minimizing CQ and PFH leakage (**Figure** **4**A). Energy dispersive spectroscopy (EDS) elemental mapping validated the presence of Fe, Cu, S, F, C, N, and O, confirming the successful fabrication of the hybrid nanomaterial (Figure 4B; Figure S14, Supporting Information). DLS measurements revealed a hydrodynamic diameter of 145.5 nm with a polydispersity index (PDI) of 0.062 (Figure 4C). Zeta potential analysis indicated successful surface modifications, with values of −3.55 mV for FCM, −17.10 mV for FCMP, −19.39 mV for FCMP@CQ, and −17.15 mV for FCMP@CQ/PFH (Figure 4D). UV–vis spectrometry further validated the encapsulation of CQ within the Fe–Cu MOFs, as evidenced by the characteristic absorption peak of CQ at 342 nm (Figure 4E). The drug loading content (LC%) of CQ was approximately 8.3%, as calculated from the UV–vis standard calibration curve (Figure S15, Supporting Information). Fourier transform infrared (FTIR) spectroscopy was performed to confirm the successful surface modification and component incorporation within FCMP@CQ/PFH nanoparticles (Figure 4F). The characteristic absorption peaks of mPEG‐NH_2_ were observed at 1091, 1461, and 2871 cm^−1^, corresponding to C─O, C─N, and ─CH_2_ stretching vibrations, respectively. For PDA, distinctive peaks at 807, 1270, 1488, and 1614 cm^−1^ were assigned to C─H out‐of‐plane bending vibration, C─N stretching vibration, C─C and C═C stretching vibrations, respectively.^[^ ^33^ ^]^ CQ exhibited prominent peaks at 1607 and 517 cm^−1^. The FTIR spectrum of FCMP@CQ/PFH retained these key absorption bands, indicating the successful integration of PDA, CQ, and PEG moieties into the final nanoplatform. Stability assays demonstrated that FCMP@CQ/PFH maintained its structural integrity across various buffer media for one week (Figure 4G), indicating its potential for in vivo applications. Considering the presence of GSH‐responsive disulfide bonds within the framework, we hypothesized that the reductive tumor microenvironment could induce GSH‐mediated cleavage and subsequent structural disassembly of the FCMP@CQ/PFH nanoparticles. To validate this, the nanoparticles were incubated in PBS with or without 10 mM GSH to mimic reductive conditions. As anticipated, a time‐dependent degradation of the MOFs was observed in the GSH‐containing group, whereas no appreciable morphological changes were detected in the absence of GSH, as confirmed by TEM images (Figure 4H; Figure S16, Supporting Information). We next tested the pH/GSH‐responsive and ultrasound‐triggered drug release capability of FCMP@CQ/PFH. The controlled release profiles of CQ from FCMP@CQ/PFH were assessed at pH 7.4 and pH 6.5, with or without 10 mmol L^−1^ GSH, mimicking the acidic TME. CQ release was significantly enhanced in the presence of GSH, reaching over 88% at pH 6.5 and 70% at pH 7.4 after 72 h, compared to 61% and 53%, respectively, without GSH (Figure 4I). The slightly elevated release at pH 6.5 compared to pH 7.4 highlights the system's pH‐responsive nature, likely attributed to weakened electrostatic interactions between FCMP@CQ/PFH and CQ in acidic environments. Ultrasound exposure further amplified CQ release, particularly in the acidic TME, achieving a release rate of 93% at pH 6.5, facilitated by the cavitation effect of perfluorocarbon microbubbles.^[^ ^34^ ^]^ To evaluate the ultrasound imaging ability of FCMP@CQ/PFH, in vivo and in vitro imaging experiments were carried out with saline as a negative control. As shown in Figure 4J, the US images demonstrated significant signal enhancement following US exposure, both in vitro and in vivo. These results underscore the theranostic potential of FCMP@CQ/PFH, combining therapeutic and diagnostic functionalities for cancer treatment.

### 2.5 Therapeutic Efficacy and Immunotherapy Capabilities of FCMP@CQ/PFH In Vitro

**Figure 5:**
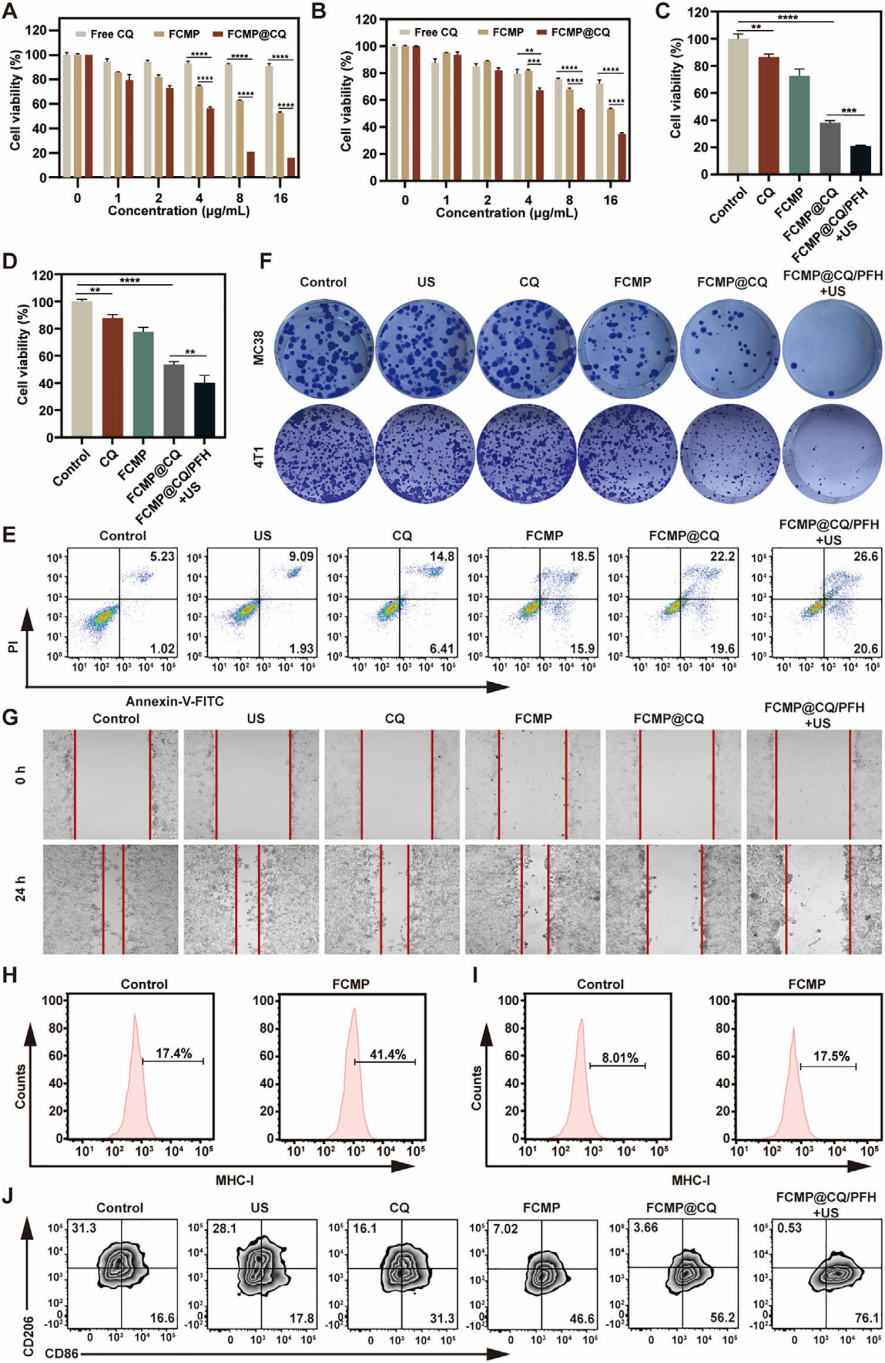
Therapeutic efficacy of NPs and immunotherapy capabilities in vitro. A) Cell viability of MC38 cells treated with free CQ, FCMP, and FCMP@CQ for 24 h. B) Cell viability of 4T1 cells treated with free CQ, FCMP, and FCMP@CQ for 24 h. C) Cell viability of MC38 cells after different treatments for 24 h. D) Cell viability of 4T1 cells after different treatments for 24 h. E) Flow cytometric analysis of cell apoptosis by Annexin V‐FITC/PI of MC38 cells after different treatments for 24 h. F) Colony formation assay images of 4T1 cells and MC38 cells after different treatments for 12 days at the indicated conditions. G) Cell migration inhibition of 4T1 cells after various treatments for 24 h. H) Flow cytometry detection of MHC‐I expression in MC38 tumor cells after treatment with 25 µg mL^−1^ FCMP for 24 h. I) Flow cytometry detection of MHC‐I expression in 4T1 tumor cells after treatment with FCMP. J) Flow cytometric analysis of M1‐like (CD86^+^) and M2‐like macrophages (CD206^+^) gated on CD11b^+^F4/80^+^ cells after treatment of M2‐polarized BMDMs with different formulations for 24 h. All data are expressed as mean ± SD (*n* = 3). Statistical significance was determined by one‐way ANOVA with Tukey's test. ^*^p < 0.05, ^**^p < 0.01, ^***^p < 0.001, ^****^ *p* < 0.0001.

We then systematically evaluated the therapeutic potential and immunomodulatory capabilities of FCMP@CQ/PFH, focusing on its role as an autophagy inhibitor and its ability to enhance synergistic effects with low‐dose CQ. Initially, we examined the impact of FCMP@CQ/PFH on tumor cell autophagy. Western blot analysis (Figures S17 and S18, Supporting Information) revealed significant accumulation of LC3‐II and p62 following FCMP@CQ/PFH treatment, indicating a pronounced autophagy‐inhibitory effect surpassing that of free CQ or FCMP alone. This suggests that the disruption of autophagic flux, likely through impediment of autophagosome‐lysosome fusion, contributes to enhanced anticancer efficacy. To validate the synergistic effects of FCMP@CQ/PFH, the cytotoxicity of free CQ, FCMP, and FCMP@CQ was assessed in 4T1 and MC38 cancer cells using the CCK‐8 assay. Free CQ exhibited minimal cytotoxicity, while FCMP induced only slight tumor cell death. In contrast, FCMP@CQ demonstrated significantly higher tumoricidal activity, particularly at a CQ concentration of 4 µg mL^−1^, confirming its robust combined therapeutic efficacy (**Figure** **5**A,B). This enhanced effect was attributed to the augmented drug release facilitated by ultrasound. Importantly, US treatment alone (1 MHz, 2 W cm^−^^2^ for 30–60 s) did not significantly affect cell viability, confirming its safety for in vivo applications (Figure S19, Supporting Information). Further investigations using CCK‐8 assays highlighted the superior antiproliferative effects of FCMP@CQ/PFH + US compared to other treatments. After 24 h, FCMP@CQ/PFH + US exhibited the most pronounced reduction in cancer cell viability (Figure 5C,D). In contrast, FCMP@CQ/PFH demonstrated excellent biocompatibility with minimal cytotoxicity against normal cell lines, including HUVEC and MCF10A cells. Cell viability remained consistently high (>80%) even at CQ‐equivalent concentrations up to 16 µg mL^−1^ (Figure S20, Supporting Information), confirming the superior biosafety profile of FCMP@CQ/PFH‐mediated therapeutic intervention.

Flow cytometry analysis of Annexin V/PI staining corroborated these findings, showing that apoptosis rates in MC38 cells treated with FCMP@CQ/PFH + US reached 47.2%, significantly exceeding the rates observed with free CQ (21.2%) and FCMP (34.4%) (Figure 5E). Similarly, in 4T1 cells, the combination therapy with FCMP@CQ/PFH + US achieved the highest apoptosis rates among all treatment groups (Figure S21, Supporting Information), underscoring its potential for effective cancer treatment. These results suggest that autophagy inhibition plays a pivotal role in promoting tumor cell apoptosis, with promising implications for biomedical applications. Consistent with these findings, colony formation assays demonstrated that FCMP@CQ/PFH + US effectively eradicated tumor cell colonies even at minimal concentrations, highlighting its long‐term cytotoxicity (Figure 5F). Additionally, wound‐healing assays in 4T1 and MC38 cells demonstrated that the combination treatment significantly suppressed tumor cell migration compared to free CQ or FCMP alone (Figure 5G, Figure S22, Supporting Information), reinforcing the role of autophagy inhibition in attenuating tumor cell dissemination and enhancing antimetastatic efficacy.

To explore the immunomodulatory properties of FCMP@CQ/PFH, we investigated its effects on MHC‐I expression in 4T1 and MC38 cells. Flow cytometry analysis revealed a marked increase in MHC‐I^+^ cells following FCMP treatment, with 41.4% of MC38 and 17.5% of 4T1 cells expressing MHC‐I, compared to 17.4% and 8.01% in PBS‐treated controls, respectively (Figure 5H,I; Figures S23 and S24, Supporting Information). This represents a 2.38‐ and 2.18‐fold increase, highlighting the capacity of FCMP to enhance antigen presentation and immune recognition. Given the critical role of TAMs in tumor progression and immune suppression, we further examined whether FCMP@CQ/PFH could modulate TAM polarization. Flow cytometry analysis of bone marrow‐derived macrophages (BMDMs) demonstrated that FCMP@CQ/PFH + US treatment significantly upregulated CD86 expression (a marker of M1 macrophages) while downregulating CD206 expression (a marker of M2 macrophages) compared to other groups (Figure 5J; Figure S25, Supporting Information). This pronounced shift from the tumor‐supportive M2 phenotype to the tumoricidal M1 phenotype suggests a reprogramming of the tumor immune microenvironment, thereby overcoming immunosuppression and enhancing antitumor immunity. Collectively, these findings provide compelling evidence that FCMP@CQ/PFH, particularly in combination with ultrasound, effectively suppresses autophagy, induces tumor cell apoptosis, impairs migration, and reprograms the tumor immune microenvironment, offering a promising strategy for cancer immunotherapy.

### 2.6 Antitumor Efficacy of FCMP@CQ/PFH in a Murine Colorectal Cancer Model

**Figure 6:**
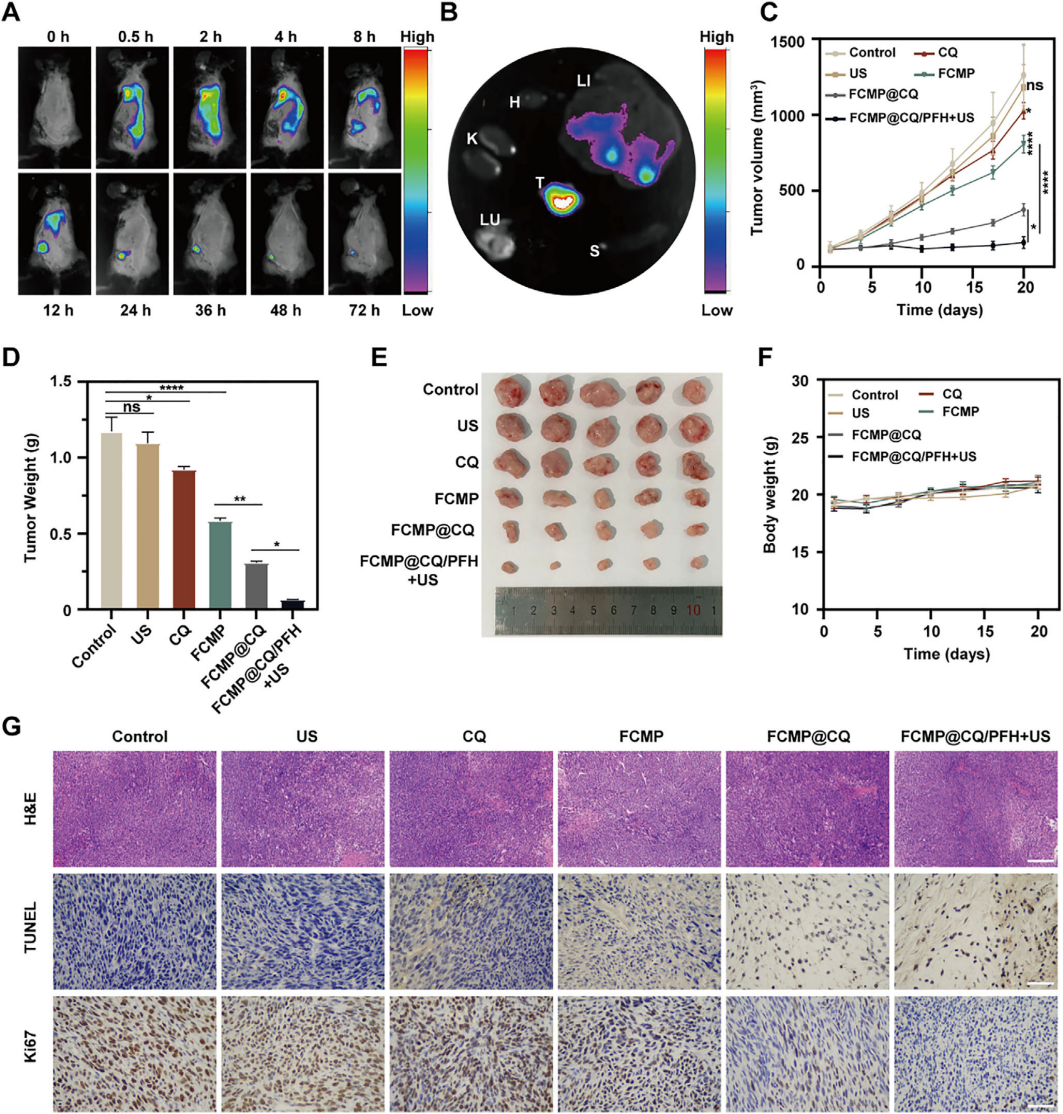
Evaluation of the antitumor effect of NPs in a murine colorectal cancer model. A) In vivo fluorescence images of MC38 tumor‐bearing mice at different time points after i.v. injection of Cy5.5‐FCMP@CQ/PFH. B) Ex vivo fluorescence images of tumor (T) and major organs including heart (H), liver (Li), spleen (S), lung (Lu), and kidney (K) collected at 24 h post‐injection. C) Tumor growth curves of mice from different groups. D) Average tumor weights after the intravenous administration of different materials. E) Photograph of tumors collected from sacrificed mice after different treatments. F) Body weight changes of tumor‐bearing mice that were treated with different groups over the treatment process. G) Representative images of H&E, TUNEL and Ki67 staining of resected tumor tissues from mice that were administered different treatments. Scale bar: 100 µm. All data are expressed as mean ± SD (*n* = 5). Statistical significance was determined by one‐way ANOVA with Tukey's test. ^*^p < 0.05, ^**^p < 0.01, ^***^p < 0.001, ^****^ *p* < 0.0001 and ns, no significant difference.

To evaluate the therapeutic efficacy of FCMP@CQ/PFH in vivo, we first investigated the biodistribution of the NPs in an MC38 tumor‐bearing mouse model. The accumulation of Cy5.5‐labeled FCMP@CQ/PFH at the tumor site was monitored through luminescence imaging in live animals and excised organs. Notably, a time‐dependent increase in NP accumulation was observed at the tumor site from 8 h post‐injection, reaching a peak at 24 h (**Figure** **6**A), highlighting the robust tumor‐targeting capability of FCMP@CQ/PFH. Ex vivo fluorescence imaging further confirmed significantly higher fluorescence intensity in tumors at 24 h compared to other organs (heart, liver, spleen, lung, and kidney), suggesting prolonged systemic circulation and tumor‐specific accumulation of the FCMP@CQ/PFH (Figure 6B). As a prelude to the tumor therapeutic study, we conducted preliminary evaluation of the pharmacokinetics of FCMP@CQ/PFH. The blood retention profiles for Fe and Cu are shown in Figure S26, Supporting Information.

Encouraged by the potent antitumor activity demonstrated in vitro, we proceeded to assess the therapeutic efficacy of FCMP@CQ/PFH in vivo using subcutaneous MC38 tumor‐bearing mice. When tumor volumes reached approximately 100 mm^3^, mice were randomly assigned to six treatment groups: control (PBS), US alone, free CQ, FCMP, FCMP@CQ, and FCMP@CQ/PFH + US. Intravenous administration was performed every three days for a total of four doses, with or without ultrasound exposure, following the outlined treatment regimen (Figure S27, Supporting Information). Tumor volumes were measured every three days, and upon completion of treatment, tumors were excised for imaging and weight analysis. As shown in Figure 6C, tumor growth was significantly suppressed in the FCMP@CQ group compared to the CQ and FCMP groups, indicating a synergistic antitumor effect. However, CQ and FCMP alone exhibited only moderate tumor inhibition. Notably, the FCMP@CQ/PFH + US group achieved near‐complete tumor eradication with minimal recurrence observed throughout the treatment period, indicating the potent therapeutic efficacy of the combined strategy. Consistent with these findings, tumor weights at the experimental endpoint further confirmed the superior antitumor activity of FCMP@CQ/PFH + US (Figure 6D,E). To further elucidate the therapeutic effects at the histological level, excised tumors were subjected to hematoxylin and eosin (H&E), terminal deoxynucleotidyl transferase–mediated deoxyuridine triphosphate nick end labeling (TUNEL), and Ki67 staining (Figure 6G). H&E staining revealed significantly greater tumor tissue damage in the FCMP@CQ/PFH + US group compared to the PBS control, while TUNEL staining demonstrated enhanced apoptosis in this treatment group, aligning with the observed tumor regression. Furthermore, Ki67 immunohistochemical analysis, which assesses cell proliferation, showed a pronounced reduction in Ki67‐positive cells in tumors treated with FCMP@CQ/PFH + US, indicating effective suppression of tumor cell proliferation.

Furthermore, body weight was monitored throughout the treatment course, with no significant differences observed across groups, indicating minimal systemic toxicity (Figure 6F). Histopathological analysis (H&E staining) of major organs (heart, liver, spleen, lung, and kidney), along with serum biochemical markers—including alanine aminotransferase (ALT), aspartate aminotransferase (AST), creatinine and urea—revealed no detectable abnormalities (Figures S28 and S29, Supporting Information), confirming the biosafety of FCMP@CQ/PFH. Taken together, these findings demonstrate the exceptional tumor‐targeting ability, robust therapeutic efficacy, and favorable biocompatibility of FCMP@CQ/PFH, particularly in combination with ultrasound, highlighting its promise for future translational applications.

### 2.7 Autophagic Flux Blockade, Polarization of TAMs and Immune Response Activation Induced by FCMP@CQ/PFH in the MC38 Tumor‐Bearing Mouse Model

**Figure 7:**
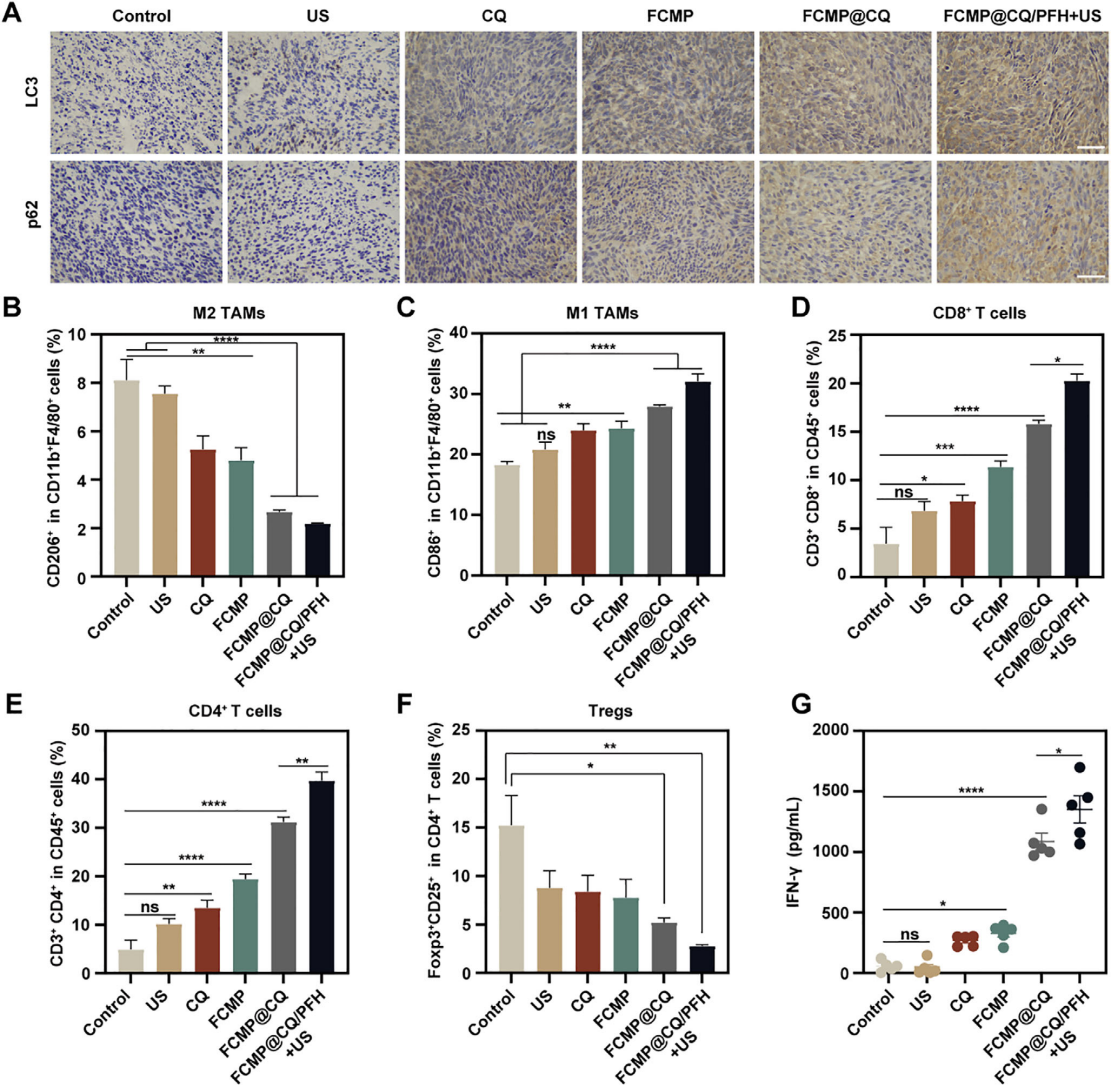
Autophagy inhibition and immune activation of the NPs in the MC38 tumor‐bearing mouse model. A) Immunohistochemistry of tumor biopsies stained with LC3 and p62 after different treatments. Scale bar: 100 µm. B,C) Flow cytometric analysis of the relative quantification of M2‐like macrophages (CD206^+^) and M1‐like macrophages (CD86^+^) gated on F4/80^+^CD11b^+^CD45^+^ cells. D) Flow cytometric analysis of CD3^+^CD8^+^ T cells infiltrated in the tumor gating on CD45^+^ cells. E) Flow cytometric analysis of CD3^+^CD4^+^ T cells infiltrated in the tumor gating on CD45^+^ cells. F) Relative quantification of Treg (Foxp3^+^ CD25^+^ gated on CD4^+^ CD45^+^) cells in tumor tissue from mice with different treatments. Data are expressed as mean ± SD (*n* = 4). G) Immune cytokines (IFN‐γ) tested by ELISA kit in the serum acquired from mice in various treatment groups (*n* = 5; mean ± SD). Statistical significance was determined by one‐way ANOVA with Tukey's test. ^*^p < 0.05, ^**^p < 0.01, ^***^p < 0.001, ^****^ *p* < 0.0001 and ns, no significant difference.

To explore the underlying mechanism of autophagy in the antitumor response, LC3 and p62 immunohistochemistry were performed. The results demonstrated a significant upregulation of both LC3 and p62 expression in the FCMP@CQ/PFH + US group compared to the PBS control, suggesting increased autophagy accompanied by impaired autophagic flux (**Figure** **7**A). Given that autophagy can facilitate tumor progression through metabolic and immune regulatory mechanisms, accumulating evidence indicates that autophagic flux blockade can enhance immunotherapy efficacy.^[^ ^35^ ^]^ To further investigate the impact of FCMP@CQ/PFH + US treatment on the tumor immune microenvironment, flow cytometry analysis was performed post‐treatment. The results revealed a significant reduction in the proportion of M2‐type TAMs and a concurrent increase in M1‐type TAMs in both the FCMP@CQ and FCMP@CQ/PFH + US groups, compared to the control and US‐only groups. These findings suggest that FCMP@CQ/PFH + US effectively promotes TAM repolarization from the immunosuppressive M2 phenotype to the antitumor M1 phenotype (Figure 7B,C). Subsequently, the potential of FCMP‐mediated MHC‐I restoration to enhance antitumor cytotoxicity was examined. Flow cytometry analysis demonstrated a significantly higher proportion of CD3^+^ T cells (CD3^+^CD45^+^) in the FCMP@CQ/PFH + US group relative to other treatment groups (Figure S30, Supporting Information). Furthermore, the highest levels of CD8^+^ and CD4^+^ T cell infiltration were observed in the FCMP@CQ/PFH + US‐treated tumors, with FCMP@CQ alone also promoting greater T cell infiltration compared to free CQ or FCMP alone (Figure 7D,E). These findings support the hypothesis that TAM polarization and enhanced T cell infiltration contribute to improved therapeutic efficacy. Given the opposing immune functions of CTLs and regulatory T cells (Tregs), the impact of FCMP@CQ/PFH + US treatment on Tregs was also evaluated. Flow cytometry analysis revealed a significant reduction in the proportion of Foxp3^+^CD25^+^CD4^+^ Tregs in tumors from the FCMP@CQ/PFH + US group compared to the PBS control (Figure 7F). Consistent with this immune activation, enzyme‐linked immunosorbent assay (ELISA) results showed that the highest levels of interferon‐gamma (IFN‐γ) were detected in the FCMP@CQ/PFH + US group (Figure 7G). In conclusion, FCMP@CQ/PFH + US treatment effectively induces autophagic flux blockade, promotes TAM polarization toward the M1 phenotype, and enhances immune activation, which may collectively contribute to its superior antitumor efficacy.

### 2.8 Antitumor Efficacy and Immune Response of FCMP@CQ/PFH in an Orthotopic Breast Cancer Model

**Figure 8:**
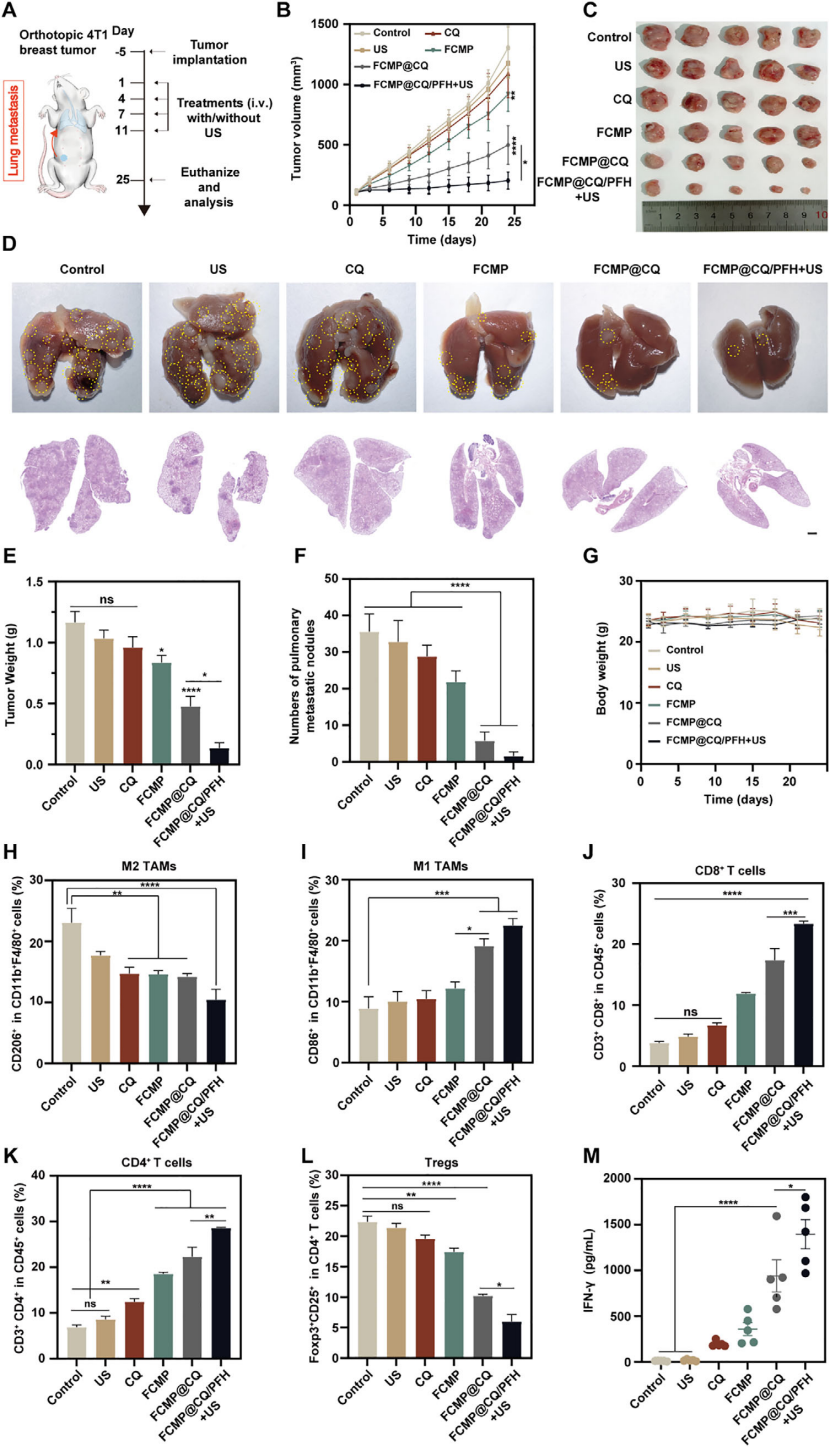
Cancer treatment and anti‐tumor immune response of NPs using an orthotopic breast tumor model. A) Schematic of the orthotopic 4T1 breast cancer mouse model and treatment protocol. B) The tumor growth kinetics of tumor‐bearing mice that were intravenously injected with different treatments (*n* = 5; mean ± SD). C) Tumor images collected from sacrificed mice after different treatments (*n* = 5). D) Representative images and H&E staining showing metastatic nodules (labeled with circles) in resected lungs from tumor‐bearing mice at the end of treatment with different groups, and F) quantified pulmonary metastatic nodules (*n* = 5; mean ± SD). Scale bar: 1 mm. E) Average tumor weights of excised primary tumors from mice at the end of different treatments (*n* = 5; mean ± SD). G) Body weight changes of tumor‐bearing mice treated with different groups over the treatment process (*n* = 5). H, I) Flow cytometry quantification of M2‐like macrophages (CD206^+^) and M1‐like macrophages (CD86^+^) gating on F4/80^+^CD11b^+^CD45^+^ cells (*n* = 4; mean ± SD). J) Quantitative analysis of the percentage of CD3^+^CD8^+^ T cells infiltrated in the tumor gating on CD45^+^ cells (*n* = 4; mean ± SD). K) Relative quantification of CD3^+^CD4^+^ T cells in the tumor gating on CD45^+^ cells (*n* = 4; mean ± SD). L) Relative quantification of Treg (Foxp3^+^CD25^+^ gated on CD4^+^CD45^+^) cells in tumor tissue from mice with different treatments (*n* = 4; mean ± SD). M) Immune cytokines (IFN‐γ) tested by ELISA kit in the serum acquired from mice in various treatment groups (*n* = 5; mean ± SD). Statistical significance was determined by one‐way ANOVA with Tukey's test. ^*^p < 0.05, ^**^p < 0.01, ^***^p < 0.001, ^****^ *p* < 0.0001 and ns, no significant difference.

Encouraged by the robust therapeutic efficacy observed in the immunogenic MC38 tumor model, we further evaluated the potential of FCMP@CQ/PFH in an orthotopic breast cancer model to assess its impact on tumor growth and metastasis (**Figure** **8**A). To establish the model, 4T1 cells were injected into the mammary fat pad of BALB/c mice, followed by treatment with different therapeutic regimens. Tumor progression and body weight were monitored throughout the study. Upon completion of the treatment course, primary tumors were excised for imaging and weight measurement. Tumor growth curves and representative images demonstrated that the US‐only and free CQ groups exhibited tumor progression comparable to the PBS control. In contrast, the FCMP@CQ/PFH + US group exhibited the most significant inhibition of primary tumor growth, yielding the smallest tumor volume and weight, whereas treatment with FCMP alone resulted in a moderate tumor‐suppressive effect (Figure 8B,C,E). To further assess the potential of FCMP@CQ/PFH in mitigating metastatic spread, pulmonary metastases were analyzed at the study endpoint. Histological examination and imaging of lung tissues revealed substantial metastatic burden in the control, US‐only, and free CQ groups, while mice treated with FCMP@CQ and FCMP@CQ/PFH + US exhibited a marked reduction in lung metastases. Notably, the FCMP group demonstrated a modest suppressive effect on metastatic dissemination (Figure 8D,F). Furthermore, evaluation of systemic toxicity showed no significant changes in body weight across all treatment groups, underscoring the biosafety of the nanoparticle‐based therapy (Figure 8G). Collectively, these findings highlight the potent therapeutic potential of FCMP@CQ/PFH + US in treating metastatic breast cancer and underscore its promise in cancer immunotherapy.

To further elucidate the immune mechanisms underlying the antitumor effects of FCMP@CQ/PFH + US, TAM populations were analyzed by flow cytometry. The results demonstrated a significant increase in the proportion of M1‐type macrophages, accompanied by a reduction in M2‐type macrophages following FCMP@CQ/PFH + US treatment compared to the control group (Figure 8H,I), indicating effective TAM repolarization toward a proinflammatory, antitumor phenotype. In addition, effector T cell activation was evaluated. FCMP@CQ/PFH + US treatment resulted in the highest infiltration of CD3^+^ T cells within the tumor microenvironment, surpassing all other treatment groups (Figure S31, Supporting Information). Further characterization of T cell subsets revealed that both CD4^+^ and CD8^+^ T cell populations were significantly enriched in tumors treated with FCMP@CQ and FCMP@CQ/PFH + US compared to the control and US‐only groups (Figure 8J,K). Given the immunosuppressive role of Tregs in the tumor microenvironment, we also examined their infiltration levels post‐treatment. Flow cytometric analysis revealed that the FCMP@CQ and FCMP@CQ/PFH + US groups significantly decreased the proportion of Tregs, thereby alleviating immune suppression and facilitating an enhanced antitumor response (Figure 8L). Furthermore, ELISA quantification of IFN‐γ, a key proinflammatory cytokine, demonstrated a pronounced increase in IFN‐γ secretion from the FCMP@CQ/PFH + US group compared to all other groups (Figure 8M). These results demonstrate that FCMP@CQ/PFH + US treatment exerts potent immunomodulatory effects by stimulating cytotoxic and effector T cells, promoting M2‐to‐M1 macrophage repolarization, and reducing Treg‐mediated immunosuppression, collectively contributing to enhanced antitumor immunity.

## 3 Conclusion

In summary, we have successfully engineered a redox‐enzyme‐integrated nanoplatform, FCMP@CQ/PFH, which is designed to integrate US‐mediated therapy with autophagic flux blockade and immune activation. By harnessing the intrinsic multienzyme‐like activities of Fe–Cu MOFs, this system induces oxidative stress and redox imbalance, effectively disrupting autophagy within tumor cells. The co‐delivery of CQ further amplifies autophagy inhibition by preventing autophagosome‐lysosome fusion, collectively restoring MHC‐I expression and reversing immune evasion. Notably, PFH undergoes a phase transition in response to US, allowing real‐time monitoring of therapeutic efficacy. Our results demonstrate that FCMP@CQ/PFH exerts a significant synergistic antitumor effect by inhibiting autophagic flux, promoting M2‐to‐M1 macrophage polarization, enhancing cytotoxic T lymphocyte infiltration, and reducing Treg‐mediated immunosuppression, ultimately suppressing tumor progression and metastasis. These findings suggest that this rationally designed nanoplatform effectively remodels the tumor immune microenvironment, offering a promising strategy for cancer immunotherapy. Given its potent therapeutic potential, FCMP@CQ/PFH holds considerable promise as a smart nanomedicine capable of overcoming the current limitations of clinical oncology treatments.

## 4 Experimental Section

The detailed experimental methods is listed in the Supporting Information. Animals were cared for and maintained under the Guidelines of Laboratory Animals of the South China University of Technology and approved by the South China University of Technology Animal Care and Use Committee. China (approved No. 2023063).

## Conflict of Interest

The authors declare no conflict of interest.

## Supporting information



Supporting Information

## Data Availability

The data that support the findings of this study are available from the corresponding author upon reasonable request.
