# Supplementary material for: Autophagy‐Targeting Fe–Cu Nanozyme for Tumor Immune Microenvironment Remodeling and Image‐Guided Cancer Immunotherapy
Source: Adv Sci (Weinh). 2025 Oct 2;12(47):e12575. doi: 10.1002/advs.202512575 (PMC12713008; doi:10.1002/advs.202512575)
Supplement: Supplementary file 1 — Supporting Information [file ADVS-12-e12575-s001.docx]

**Supporting Information**

**Autophagy-Targeting Fe-Cu Nanozyme for Tumor Immune Microenvironment Remodeling and Image-Guided Cancer Immunotherapy**

*Li Yan, Chao Chen, Yu Liang, Xiaowan Huang, Jieying Qian, Hao Zhang, Li Zhang, Yingjia Li*, Yunjiao Zhang**

L. Yan, Y. Liang, L. Zhang, Y. Li

Department of Medicine Ultrasonics, Nanfang Hospital, Southern Medical University, Guangzhou 510515, P. R. China

Email: lyjia@smu.edu.cn

C. Chen, X. Huang, J. Qian, H. Zhang, Y. Zhang

School of Medicine, South China University of Technology, Guangzhou 510006, P. R. China

Email: zhangyunjiao@scut.edu.cn

Y. Zhang

National Engineering Research Centre for Tissue Restoration and Reconstruction and Guangdong Provincial Key Laboratory of Biomedical Engineering, South China University of Technology, Guangzhou 510006, P. R. China

**Experimental Section**

**Materials and Antibodies:** Iron (II) chloride tetrahydrate (FeCl_2_·4H_2_O), dithiodiglycolic acid, N, N-dimethylformamide (DMF) were purchased from Macklin, Shanghai, China. Cupric chloride dihydrate (CuCl_2_·2H_2_O), pyridine, triethanolamine (TEA), and dopamine hydrochloride were purchased from Aladdin, Shanghai, China. Polyvinylpyrrolidone (PVP, MW = 40,000) was from solarbio company in Beijing, China. Methoxy polyethylene glycol amine (mPEG-NH_2_, MW = 5000) was purchased from Tansh-Tech. Cyanine5.5 N-hydroxysuccinimide ester (Cy5.5-NHS ester) was bought from Shanghai Chemical Reagents Company. Chloroquine diphosphate salt (CQ), bafilomycin A1 (Baf-A1), wortmannin (Wort), reduced glutathione (GSH) and lipopolysaccharide (LPS) were obtained from Sigma-Aldrich, USA. Recombinant Murine IL-4 was purchased from PeproTech Inc (USA). Dulbecco modified Eagle medium (DMEM), fetal bovine serum (FBS), and penicillin–streptomycin were purchased from Gibco. Annexin V-FITC apoptosis detection kit and cell counting kit 8 (CCK-8) were purchased from Beyotime, Shanghai, China. Anti-LC3 antibody (NB100-2220) was obtained from Novus Biologicals. Anti-GAPDH (60004-1) and anti-β-actin (66009-1) antibodies were from Proteintech. Anti-LC3 (A19665), anti-GFP-Tag (AE012) and anti-SQSTM1/p62 (A19700) antibodies were sourced from ABclonal. Anti-MHC-I (76828S), Anti-MTOR/p-MTOR (2983S/5536S), anti-p70S6K/p-p70S6K (9202S/9205S) and anti-AKT/p-AKT (4691S/4060S) antibodies were purchased from Cell Signaling Technology. Flow cytometric antibodies and mouse IFN-γ ELISA kit were obtained from Biolegend Biological Technologic Co.Ltd.

**Preparation of MOFs (11:4, 1:1, 4:11, Fe MOFs, Cu MOFs):** Fe-Cu MOFs (11:4) (FCM) was synthesized through hydrothermal synthesis assembly approach according to a previously reported method.^[1]^ Briefly, 13 mL DMF/ethanol solution (V_DMF_/V_ethanol_ = 5/3) containing FeCl_2_⋅4H_2_O (124 μL, 50 mg/mL in DMF) CuCl_2_⋅2H_2_O (38 μL, 50 mg/mL in DMF) (the total amount of Fe^2+^ and Cu^2+^ was 42.5 μmol, with a molar ratio of Fe to Cu of 11:4 ), dithiodiglycolic acid (52 μL, 100 mg/mL in DMF), PVP (300 mg), and triethylamine (TEA, 200 μL) was added to a 50 mL Teflon-lined stainless autoclave for ultrasonic dispersion and reacted at 150 °C for 12 h. FCM was obtained and washed with ethanol for removing the excess unreactive reagent and dispersed in ddH_2_O for further use. For the synthesis of other MOF formulations, the same procedure was followed with adjusted metal precursor volumes: For Fe-Cu MOFs with 1:1 and 4:11 ratios, the total amount of Fe²⁺ and Cu²⁺ was maintained at 42.5 μmol, with molar ratios of Fe to Cu of 1:1 and 4:11, respectively. For monometallic Fe MOFs, 42.5 μmol of Fe²⁺ was used, while for Cu MOFs, 42.5 μmol of Cu²⁺ was employed. All formulations maintained identical reaction conditions.

**Preparation of FCM@CQ, FCM@CQ/PFH, FCMP@CQ/PFH:** To obtain FCM loaded CQ, adding CQ (1 mg/mL, 1 mL) into the 5 mL FCM dispersion (1 mg/mL in ddH_2_O), then the obtained solution was stirred overnight. The FCM@CQ solution was freeze drying overnight. The solid mixture was put into a 25 mL three-necked flask with rubber stoppers. A vacuum pump was used to evacuate the air in the flask before the injection of 50 μL of PFH. The PFH and FCM@CQ mixture were sonicated for 5 min in the ice-water before the injection of 5 mL of PBS and another 10 min sonication in ice-water. The FCM@CQ/PFH solution was obtained. In order to wrap the PDA nano-film on FCM@CQ/PFH, the product FCM@CQ/PFH solution was dispersed in pH 8.5 Tris buffer solution (10 × 10^−3^ M) and ultrasound. Dopamine hydrochloride (0.5 mg mL^−1^) was then added and stirred for 12 h. Then, mPEG-NH_2_ (1 mg mL^−1^) was added and reacted for another 2 h to modify the FCM@CQ/PFH with PEG. Then the product was collected and washed by ultrapure water for three times to obtain FCMP@CQ/PFH.

**Preparation of Fluorescent Cy5.5-conjugated FCMP@CQ/PFH:** Cy5.5 NHS ester and the resulting FCMP@CQ/PFH NPs were dissolved in solution (PBS, pH 8.0). The required reaction environment is continuous stirring at room temperature overnight and protected from light. Finally, the excess Cy5.5-NHS-ester in the solution was removed by ultrafiltration, and the resulting Cy5.5-conjugated FCMP@CQ/PFH NPs were stored at 4 °C in dark.

**Characterization of FCMP@CQ/PFH:** TEM images, EDS element mappings, and HAADF images information of FCMP@CQ/PFH were taken using field emission scanning electron microscope on Merlin (Zeiss, German) to characterize the morphology and composition of the FCMP@CQ/PFH. The size distribution and zeta potentials of the nanoparticles were detected by DLS (Malvern Instruments, Malvern, UK). To evaluate the stability of FCMP@CQ/PFH, NPs were dispersed in water, PBS (pH 7.4), RPMI 1640 + 10% FBS, respectively, and stored at 37 °C for a week. Size was measured and collected at predetermined time interval. The FTIR experiment was conducted on a Vertex 70 spectrometer (Bruker, Germany).

Next, the concentration of loaded CQ was determined with a UV-vis spectrometer (UV-2600, Shimadzu, Japan). The drug loading content (LC) was calculated according to the following equation: LC (%) = W_Encapsulated‑drug_/W_nanoparticles_ × 100%.

**Drug Release of FCMP@CQ/PFH:** The release of CQ in FCMP@CQ/PFH was separately detected by a UV-vis spectrometer at different pH values (6.5 and 7.4) and GSH concentrations (0 and 10 mM). Briefly, 1 mL of FCMP@CQ/PFH (CQ concentration: 0.4 mg/mL) was placed into a dialysis bag (MWCO 8000) and sonicated. Next, we immersed the dialysis bags in 15 mL of PBS solution with different pH and GSH concentrations. After that, the solution was stirred at 80 rpm at 37 °C. At certain intervals, the filtrate of various groups was collected. The medium (100 μL) was taken out for analysis with a microplate reader at 342 nm and calculated using the standard curve. Meanwhile, an equal volume of fresh release medium was supplemented. Each assay was repeated in triplicate.

**In Vitro and In Vivo US Imaging:** An Acuson Sequoia ultrasound system unit with a 18L6 transducer (Siemens Healthcare, Shanghai, China) was used to assess the US imaging of FCMP@CQ/PFH. Saline was used as the negative control. In order to better simulate clinical ultrasound imaging in vitro, we used rubber gloves to load saline and NPs and then applied an ultrasound coupling agent on the rubber gloves to observe the ultrasound imaging effect. Homemade latex film and tumor-bearing mice were used respectively as experimental models, and the US imaging was performed with a B-mode ultrasonic transducer operating at 12.5 MHz.

**Cell Culture:** The murine mammary carcinoma cell 4T1 and mouse colorectal cancer cell line (MC38) were cultured in RPMI 1640 medium. The human ovary carcinoma cell (HeLa) was cultured in DMEM medium. The DMEM medium and RPMI 1640 medium both contain 10% FBS (Gibco, USA) and 1% penicillin/streptomycin (Gibco, USA). All cells were incubated in a cell incubator at 37 ℃ and 5% CO_2_. Bone marrow-derived macrophages (BMDMs) were obtained from the tibia and femoral bone marrow of 6-8 weeks-old male C57BL/6 mice. The deposited monocytes were resuspended in new high glucose DMEM (10% FBS, 1% penicillin/streptomycin, 20 ng/mL M-CSF).

**Western Blotting:** Cells were lysed with radoimmunoprecipitation assay (RIPA) buffer containing phosphatase and protease inhibitors (Sangon). Cell lysate containing 20-100 μg protein was loaded onto tris-glycine gel, separated by electrophoresis, transferred to polyvinylidene difluoride membrane (Millipore, IPVH00010). The membranes blocked with 5% skim milk for 1 h at room temperature and then incubated primary antibody overnight at 4 ℃. After incubation, the protein bands washed with TBST (TBS supplemented with 1‰ tween-20), and then incubated with appropriate secondary antibody conjugated with HRP for 1 h at room temperature. The protein bands washed four times with TBST for 10 min each, and imaged by the imaging system (Clinx Science Instruments, China).

**Immunofluorescence:** Cells were fixed in 4% paraformaldehyde in PBS for 15 min followed by permeabilization with 0.25% Triton X-100 in PBS for 15 min at room temperature. Fixed preparations were blocked with 3% BSA in PBS for 1 h, then incubated with primary antibodies against anti- LAMP1（Santa Cruz） and anti-LC3B (ABclonal) 2 h. The stained cells were washed and incubated with Alexa Fluor-conjugated secondary antibodies (Alexa Fluor 488 goat anti-rabbit and Cy3-conjugated Goat Anti-Mouse IgG) for 1 h. 4’, 6-diamidino-2-phenylindole (DAPI) was used to stain nuclei. All steps were performed at room temperature. Images were captured using a fluorescence microscope.

**Examination of lysosomes and lysosomal activities:** MC38 cells were cultured in cell culture dishes, followed by incubation with different treatments for 24 h. Cell nuclei and lysosomes were stained with Hoechst 33342 (20 ng mL^−1^) and Lyso-Sensor Green DND-189 (1 μm, 30 min, 37 °C, 5% CO_2_), respectively. To assess cathepsin B and cathepsin D activities, cells were treated with FCMP for 24 h, followed by staining with Magic Red substrates (937; ImmunoChemistry) according to the manufacturer’s instruction.

**GFP-LC3 Dot Formation Assay and GFP-mRFP-LC3 Redistribution Evaluation:** GFP-LC3/HeLa cells were observed under fluorescence microscopy after treatment, Cells were plated onto 6-well tissue culture plates, then treated with different formulations for imaging with fluorescence microscopy. The GFP-mRFP-LC3 expressing HeLa cells were constructed by GFP-mRFP-LC3 plasmid transfection. In general, HeLa cells were seeded onto a 24-well plate (1 × 10^5^ per well) in DMEM culture medium and allowed to grow until 80% confluency. Then cells were transfected using Lipofectamine 3000 referring to the manufacturer’s instructions. For each well, 10 μg of concentrated GFP-mRFP-LC3 plasmid was added into 1.5 mL of Opti-MEM medium without antibiotics and FBS, and then 10 μL of Lipofectamine 3000 was added. The diluted plasmid and Lipofectamine 3000 were preincubated for 15 min at room temperature to ensure full combination. Subsequently, the culture containing the plasmidcationic liposome complex was introduced into each well and allowed to incubate with HeLa cells. The GFP-mRFP-LC3 -expressing cells were obtained after 4 h of incubation, followed by the addition of fresh FBS-free culture containing different formulations. After another 24 h of incubation, GFP-mRFP-LC3-HeLa cells were observed under fluorescence microscopy.

**Flow Cytometry Detection of MHC-I In Vitro:** To quantitatively evaluate the MHC-I expression in MC38 and 4T1 cells after treatments with the FCMP, the cells receiving treatment were washed, counted, resuspended in PBS, and incubated anti-mouse MHC-I-FITC antibody (BioLegend), then analyzed the portion of MHC-I positive cells by flow cytometry.

**In Vitro Cytotoxicity Assay:** Cells were seeded in the 96-well plates and cell proliferation was assessed by CCK-8 assays. Briefly, CCK-8 (#C0037, Beyotime) solution was added to each well (10 μL per well). After 2 h of incubation, the absorbance at 450 nm was measured using a spectrophotometer (Elx800, BioTek, Winooski, VT, USA).

**Cell Apoptosis Assay:** Briefly, 4T1 and MC38 cells were seeded on 12-well cell culture plates at 10^5^ cells per well. After co-incubated with different groups for 24 h, cells were collected and cultured with the Annexin V-FITC Apoptosis Detection Kit (Catalogue #C1062S, Beyotime, China) according to the manufacturer’s instructions. Finally, data were analyzed via flow cytometer.

**Colony Formation Assay:** Cells were seeded on 6-well plates and then treated as different groups. After 12 h incubation, then changed to new medium. After culture for another 12 days, colonies were fixed with methanol, stained with 0.1% of crystal violet for 15 min at room temperature. The colonies were then washed with ddH_2_O and imaged.

**Wound-healing Assay:** Cells were cultured in 6-well plates. When the cells grow to a state of fusion into a monolayer, cells were scratched gently with a 100 μL pipette tip perpendicular to the cell plane, and then cells were washed three times with sterile PBS to remove scattered cells and made the gaps left by streaking clearly visible, then replaced with fresh medium. Subsequently, the cells were treated as different treatments. The images of wound healing were photographed at 0 h and 24 h.

**Evaluation of the Repolarization of M2 Macrophages:** Bone marrow-derived macrophages (BMDMs) isolated from femurs and tibias of C57BL/6 mice were cultured in DMEM medium containing 10% bovine fetal serum (FBS), 1% Penicillin-Streptomycin Solution and M-CSF (20 ng mL^-1^) for four days. BMDMs were incubated with IL-4 (20 ng mL^-1^) for 24 h to induce M2 macrophages. M2 macrophages were then incubated with culture medium containing (1) growth medium (no treatments, as control), (2) US, (3) CQ, (4) FCMP, (5) FCMP@CQ, (6) FCMP@CQ/PFH + US for 24 h respectively. Afterward, BMDMs were harvested and stained with APC/Cyanine7 anti-mouse CD45.2, PE anti-mouse CD11b, FITC anti-mouse F4/80, APC anti-mouse CD206 and Brilliant Violet 421™ anti-mouse CD86 (all bought from BioLegend), and analyzed by flow cytometer.

**Animal Experiments:** Female BALB/c mice (6-8 weeks), C57BL/6 mice (6-8 weeks) were obtained from Beijing Vital River. All mice were housed in pathogen-free conditions and kept in a room with controlled temperature (~22 °C) and humidity (45%-60%) under a 12 h light-dark cycle. The procedures were approved by the South China University of Technology Animal Care and Use Committee.

**In Vivo Distribution of FCMP@CQ/PFH NPs:** Briefly, 100 μL of cells (5 × 10^5^) was injected into 6-8 weeks old C57BL/6 to establish subcutaneous colorectal tumor model. When the tumor volume reached approximately 200 mm³, 100 μL of Cy5-labeled FCMP@CQ/PFH NPs was injected intravenously into tumor-bearing mice. Mice were imaged with In-Vivo Xtreme (Bruker) at different time points (0, 0.5, 2, 4, 8, 12, 24, 36, 48, 72 h). The mice were sacrificed 24 h after injection, and the tumor tissues were harvested, then these organs (heart, liver, spleen, lung, kidney) and tumor were fluorescently imaged with In-Vivo Xtreme.

**Pharmacokinetics study:**  C57BL/6 mice were injected with FCMP@CQ/PFH at a dose of 2.5 mg per kg (CQ dosages) of mouse body weight. At predetermined time points (0, 0.5, 1, 2, 4, 8, 12, and 24 h), blood samples were collected from the retro-orbital plexus of the eye and then placed in heparinized tubes and centrifuged to obtain plasma. An equal volume of plasma was withdrawn, and the plasma samples were then nitrated. The Iron and copper content in the plasma were determined by ICP-MS, and the values for the various time points were normalized against that of the 0 time (approximately 3 min after injection) value.

**In Vivo Antitumor Evaluation:** Tumor-bearing mice were established by subcutaneously injecting 5 × 10^6^ of MC38 cells into C57BL/6 mice (6-8 weeks old female).When the tumor volume reached approximately 100 mm^3^, the mice were randomly divided into six groups (*n* = 5) and then were administrated with PBS, US, CQ, FCMP, FCMP@CQ, FCMP@CQ/PFH + US, respectively (ultrasound was implemented at 1 MHz and 2 W/cm^2^ for 60 s, and the CQ dosages for the mice in drug groups were equivalent to 2.5 mg/kg). Such administration was repeated every 3 days for a total of four times. The tumor volume and weight of tumor-bearing mice were monitored during the treatment course. The mice were euthanized at the end point of treatment (day 21). The tumors were resected for histological staining, including LC3, p62, H&E, TUNEL, and Ki67, to examine the anticancer effect.

The unilateral 4T1 orthotopic breast tumor model was established by injecting 5 × 10^5^ of 4T1 cells into the breast fat pad of female BALB/c mice (6-8 weeks old). When the tumor volume reached approximately 100 mm^3^, the mice were randomly divided

into six groups (*n* = 5) and were intratumorally administrated with PBS, US, CQ, FCMP, FCMP@CQ, FCMP@CQ/PFH + US respectively (ultrasound was implemented at 1 MHz and 2 W/cm^2^ for 60 s, and the CQ dosages for the mice in drug groups were equivalent to 2.5 mg/kg). The administration was repeated every 3 days for a total of four times. The tumor volume and body weight of each mouse was recorded throughout the treatment course. The mice were euthanized at the end point (day 24). The primary tumors were harvested to measure the weights and photographed. The lung tissues were harvested for pulmonary metastatic nodule statistical analysis and were photographed after fixed in 4% formalin solution. Last, H&E staining of these lung tissues was conducted.

**In Vivo Immune Response:** As mentioned above, the tumors were separated from different groups of mice. Then, we used scissors to cut the tumor to make the tumor fragments as small as possible. These fragments were digested by digestive liquid. The above liquid was transferred to another centrifuge tube and centrifuged at 400 g for 5 min at 4 °C. After that, the liquid was gradient centrifuged by using different concentrations of isotonic Percoll solution. A Pasteur tube was used to draw the cell population in the middle of the liquid, and the cells were centrifuged again under the same conditions. After centrifugation, the red blood cell (RBC) lysate was added to lyse RBCs and the remaining cells were cleaned and stained with different immunofluorescent antibodies: CD45.2-APC/Cyanine7, CD11c-PE, CD11b-BV510, F4/80-FITC, CD86-BV421, CD206-PE, CD3-FITC, CD8a-BV785, CD4-BUV563 (BD Biosciences), Foxp3-APC, and CD25-PerCP594 (all bought from BioLegend except BUV563 Rat Anti-Mouse CD4). F4/80^+^, CD86 and CD206 were used to represent M1 and M2 phenotype macrophages. CD3, Foxp3, and CD25 were used on behalf of Treg cells, and the cells stained with CD3 and CD4 or CD8a antibodies were separately CD4^+^ T cells or CD8^+^ T cells, respectively. Finally, the levels of IFN-γ in the serum was respectively measured using an ELISA kit according to the manufacturer’s instructions.

**Toxicity Study:** After treatment, orbital blood of mice was collected and the following indexes were measured by an Automatic Biochemical Analyzer (3100, Hitachi, Japan): ALT, AST, creatinine and urea. Histological changes of the major organs (including heart, liver, spleen, lung, kidney, etc.) of the treated mice were analyzed. The major organs were fixed with 4% paraformaldehyde, embedded in paraffin, sliced into 5 μm sections and stained with H&E.

**Statistical Analysis:** Quantitative data are expressed as mean ± SD or mean ± SEM, as indicated in the figure captions. Comparisons among multiple groups (when more than two groups were compared) were performed using one-way analysis of variance (ANOVA) followed by Tukey's post hoc test. Unpaired two-tailed Student's t-test was used for two-group comparisons. All statistical analyses were performed with GraphPad Prism 8.0 software. Statistical significance was defined as **p* < 0.05; ***p* < 0.01; ****p* < 0.001; *****p* < 0.0001 and ns indicates no significant difference.

**Supporting Figures**


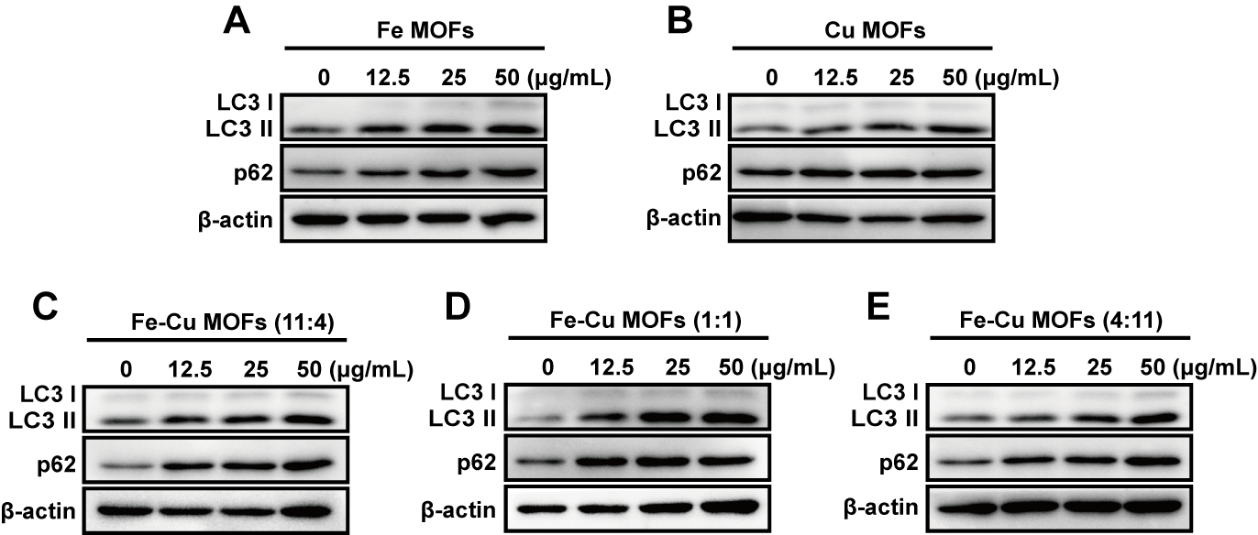


**Figure S1.** Western blot analysis of LC3 and p62 proteins in 4T1 cells treated with different concentrations of Fe MOFs (A), Cu MOFs (B), Fe-Cu MOFs at ratios of 11:4 (C), 1:1 (D), and 4:11 (E) for 24 h.


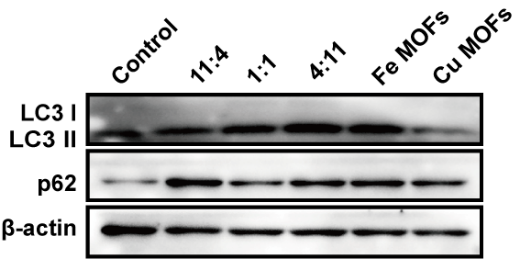


**Figure S2.** Western blot comparison of LC3 and p62 expression across all formulations at 25 μg/mL in 4T1 cells for 24 h.


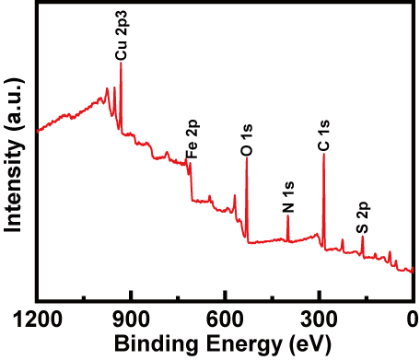


**Figure S3.** Overview of the XPS survey spectrum of MOFs (11:4).


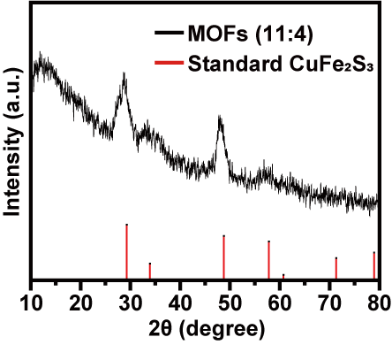


**Figure S4.** X-ray diffraction patterns (XRD) of MOFs (11:4).


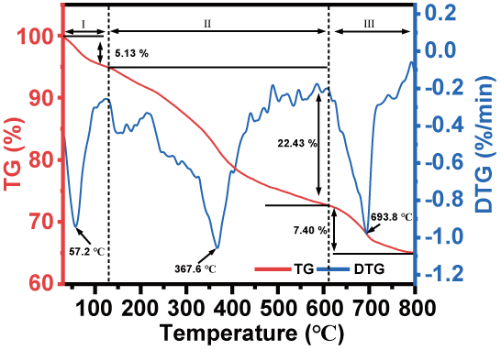


**Figure S5.** TG-DTG curves of MOFs (11:4) carried out in a nitrogen atmosphere.


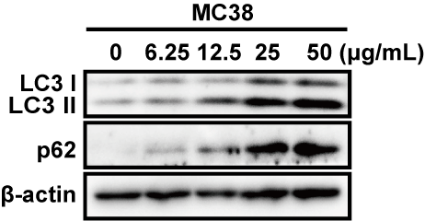


**Figure S6.** The western blotting analysis of LC3 and p62 proteins extracted from MC38 cells with the indicated treatment by different doses of FCMP for 24 h


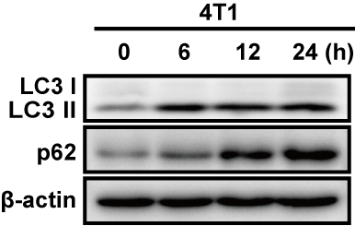


**Figure S7.** Western blotting of LC3 and p62 levels in 4T1 cells after the indicated treatment by different time of FCMP for 25 μg/mL.


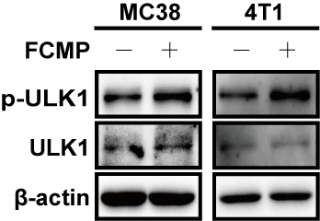


**Figure S8.** MC38 and 4T1 cells treated with PBS (control) and 25 μg/mL FCMP for 24 h analyzed by ULK1 and phospho-ULK1western blotting.


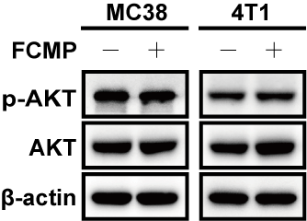


**Figure S9.** MC38 and 4T1 cells treated with PBS (control) and 25 μg/mL FCMP for 24 h analyzed by AKT and phospho-AKT western blotting.


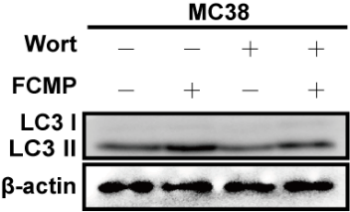


**Figure S10.** Western blotting of LC3 in MC38 cells treated with 25 μg/mL FCMP for 24 h in the presence or absence of 1 mM wortmannin (Wort).


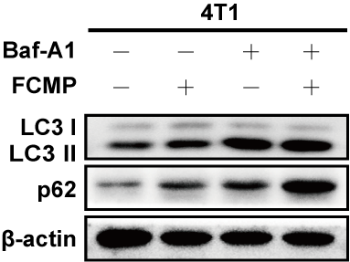


**Figure S11.** 4T1 cells were treated with 25 μg/mL FCMP for 24 h in the presence or absence of 400 nM bafilomycin A1 (added 4 h before cell harvest).


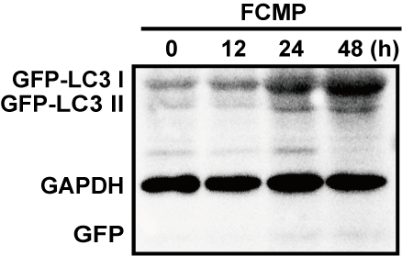


**Figure S12.** Western blotting of GFP in HeLa GFP-LC3 cells after the indicated treatment by different time of FCMP for 25 μg/mL.


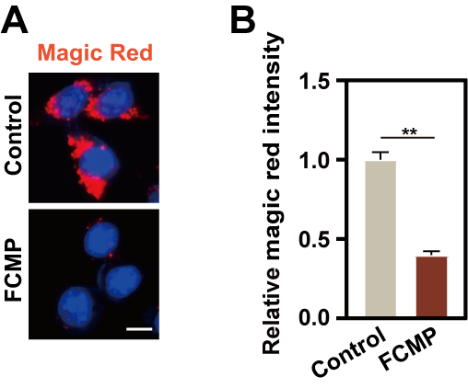


**Figure S13.** Representative fluorescence images of MC38 cells treated with 25 μg/mL of FCMP for 24 h, showing Cathepsin B activity visualized by Magic Red staining (A) and (B) quantification of Magic Red fluorescence intensity. Scale bar: 10 μm. Statistical significance was determined by unpaired two-tailed Student’s t-test. **p* < 0.05, ***p* < 0.01, ****p* < 0.001, *****p* < 0.0001.

**
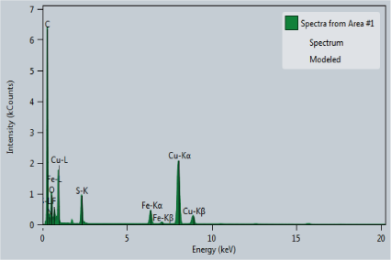
**

**Figure S14.** EDS spectrum of the obtained FCMP@CQ/PFH nanoparticles.


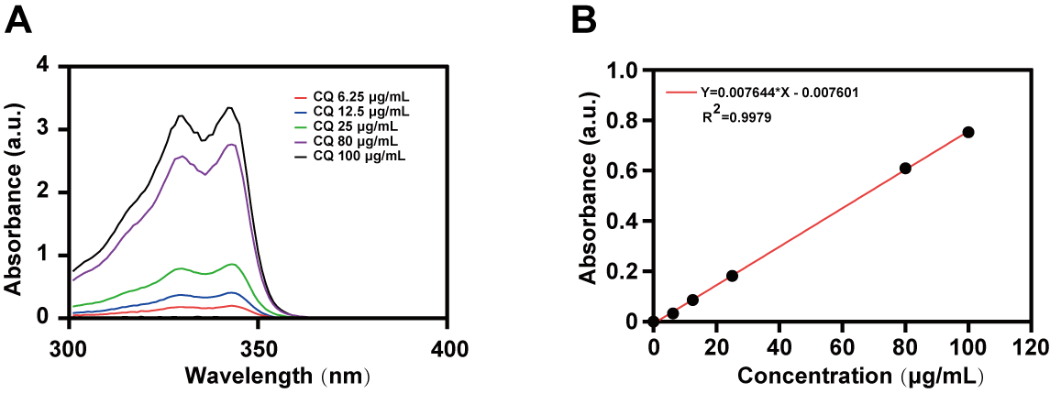


**Figure S15.** (A) UV-vis absorption spectra of CQ solution with different concentrations. (B) The standard curves of CQ solution with different concentrations at the peak of 342 nm.


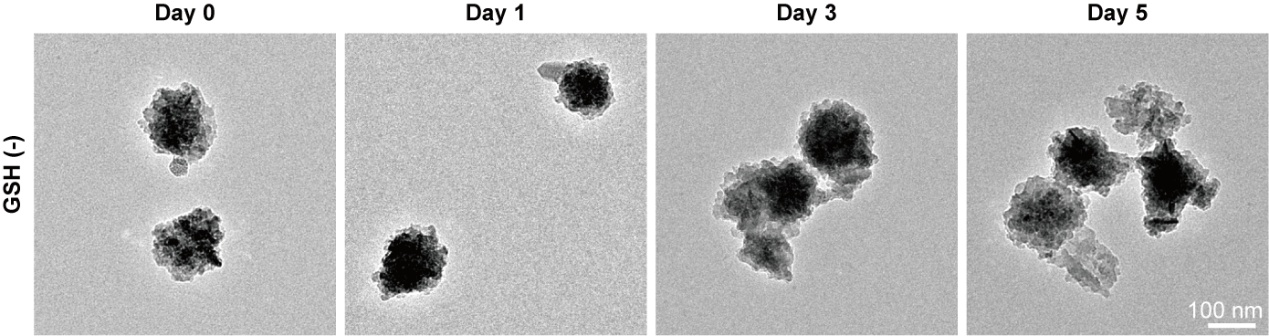


**Figure S16.** TEM images of biodegradable FCMP@CQ/PFH stored in PBS without 10

mM GSH for 0, 1, 3 and 5 days.


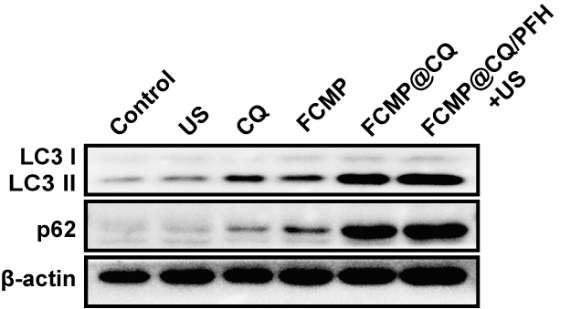


**Figure S17.** LC3 and p62 proteins level of MC38 cells after treatment with different groups for 24 h.


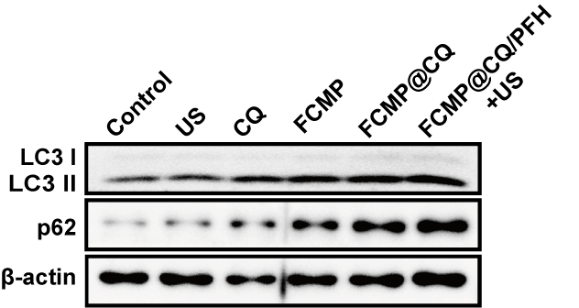


**Figure S18.** LC3 and p62 proteins level of 4T1 cells after treatment with different groups for 24 h.


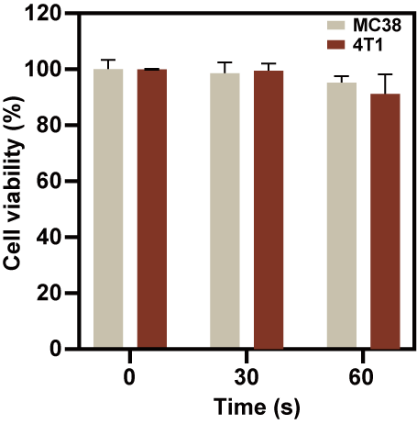


**Figure S19.** 4T1 and MC38 cells viability after treated with various time of ultrasound therapeutic apparatus via CCK8 assay.


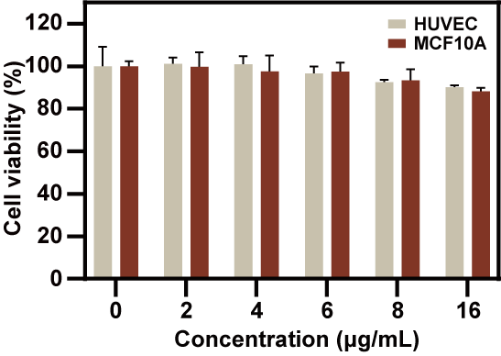


**Figure S20.** Viability of HUVEC and MCF10A cells after being treated with FCMP@CQ/PFH NPs at different CQ concentrations.


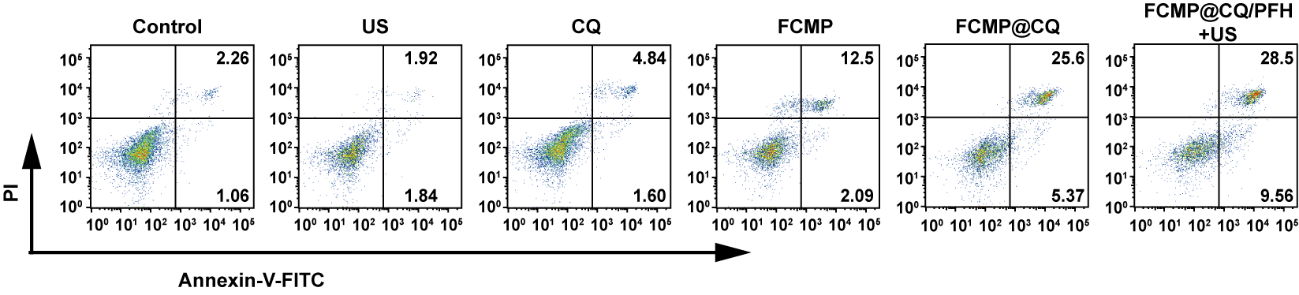


**Figure S21.** Flow cytometric analysis of cell apoptosis by Annexin V FITC/PI of 4T1 cells after different treatments for 24 h.

**
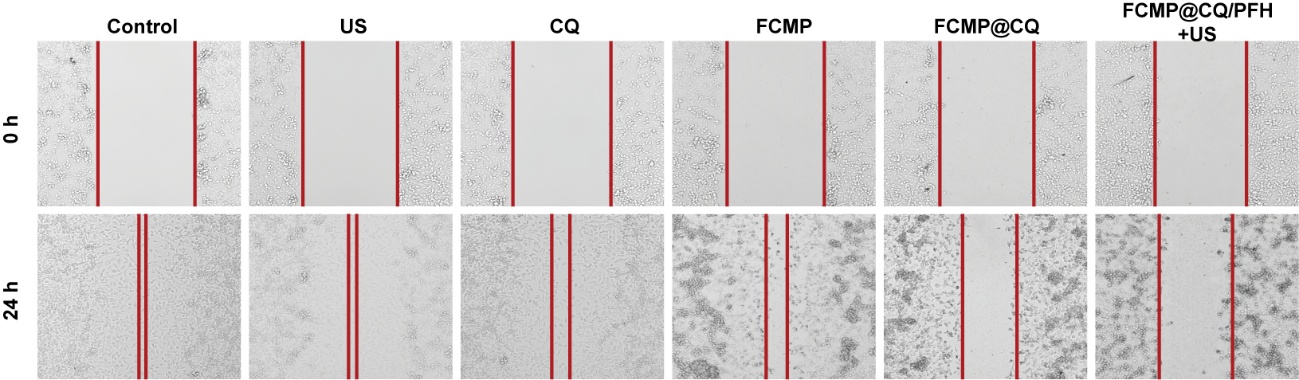
**

**Figure S22.** Cell migration inhibition of MC38 cells after various treatments for 24 h.

**
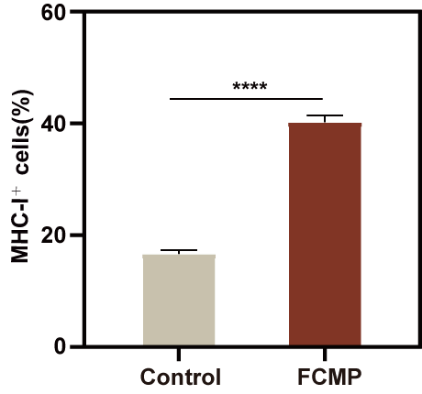
**

**Figure S23.** The quantitative analysis of MHC-I expression in MC38 tumor cells after treating with FCMP (*n* = 3; mean ± SD). Statistical significance was determined by unpaired two-tailed Student’s t-test. **p* < 0.05, ***p* < 0.01, ****p* < 0.001, *****p* < 0.0001.


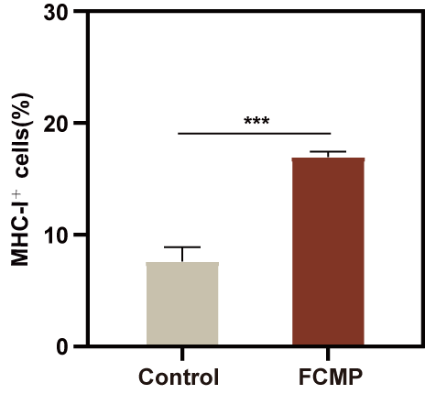


**Figure S24.** The quantitative analysis of MHC-I expression in 4T1 tumor cells after treating with FCMP (*n* = 3; mean ± SD). Statistical significance was determined by unpaired two-tailed Student’s t-test. **p* < 0.05, ***p* < 0.01, ****p* < 0.001, *****p* < 0.0001.


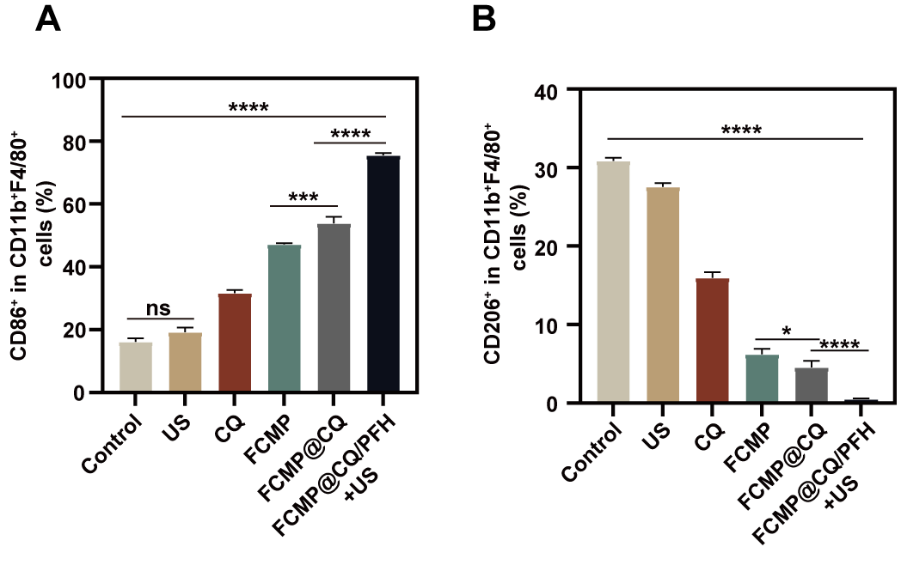


**Figure S25.** The relative quantification of M1-like macrophages (CD86^+^) (A) and M2-like macrophages (CD206^+^) (B) gating on F4/80^+^CD11b^+^cells (*n* = 3; mean ± SD). Statistical significance was determined by one-way ANOVA with Tukey’s test. **p* < 0.05, ***p* < 0.01, ****p* < 0.001, *****p* < 0.0001 and ns, no significant difference.


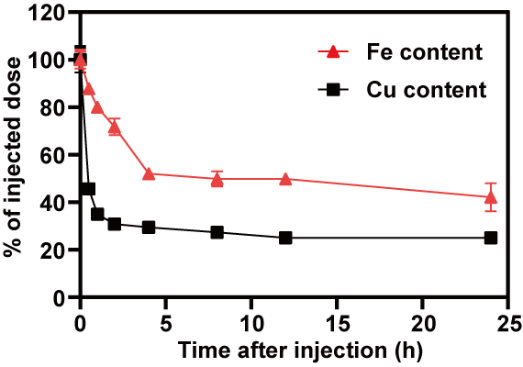


**Figure S26.** Pharmacokinetic profile of FCMP@CQ/PFH (n = 3; Mean ± SEM).


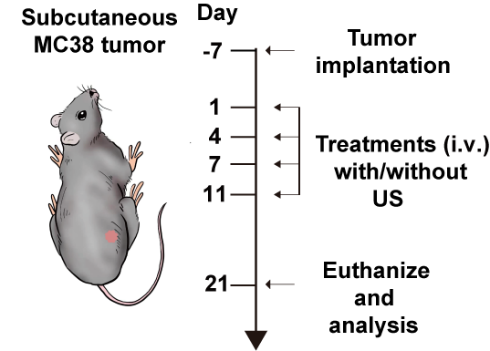


**Figure S27.** Schematic of the mouse model that bears subcutaneous MC38 colorectal tumor and the following treatment protocol.


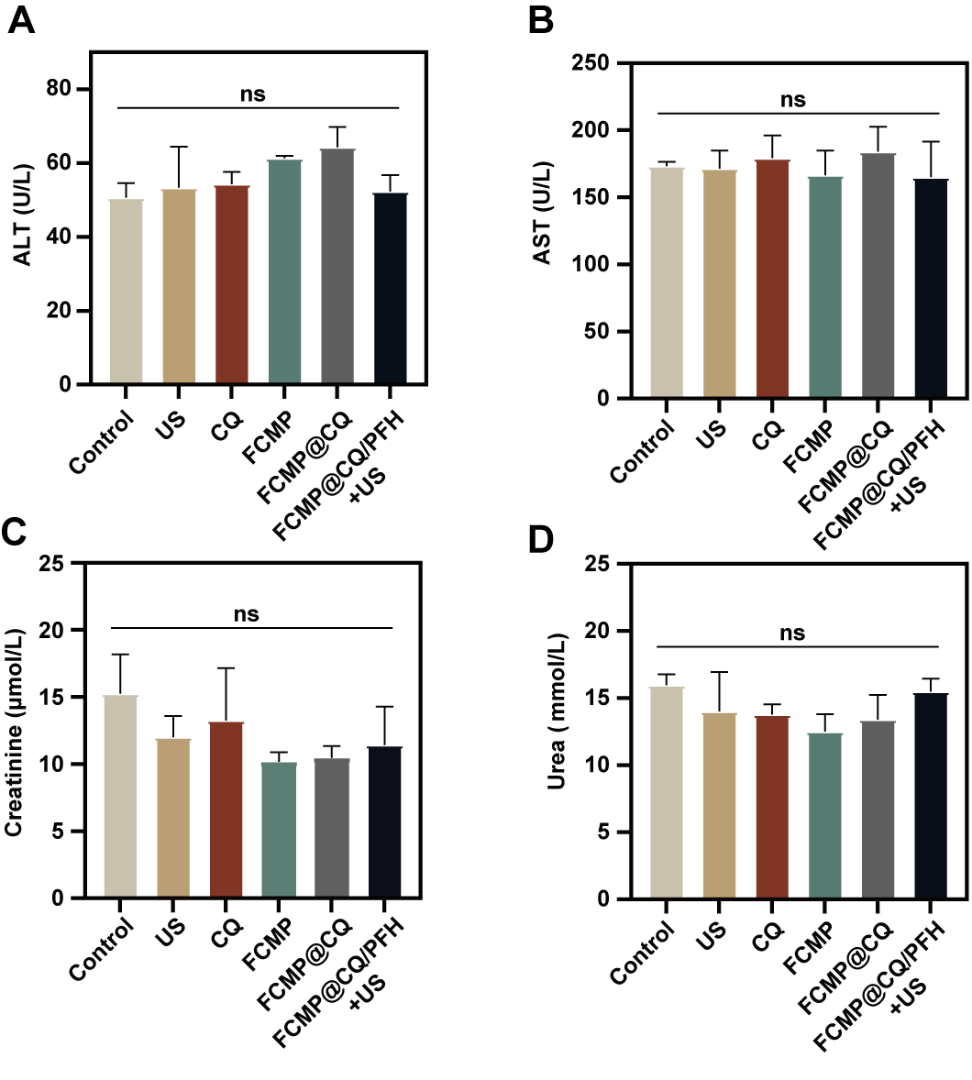


**Figure S28.** Blood biochemical analysis including levels of (A) alanine aminotransferase (ALT), (B) aspartate aminotransferase (AST) (C) creatinine and (D) urea. Data are represented as mean ± SD (n = 3). Statistical significance was determined by one-way ANOVA with Tukey’s test. **p* < 0.05, ***p* < 0.01, ****p* < 0.001, *****p* < 0.0001 and ns, no significant difference.


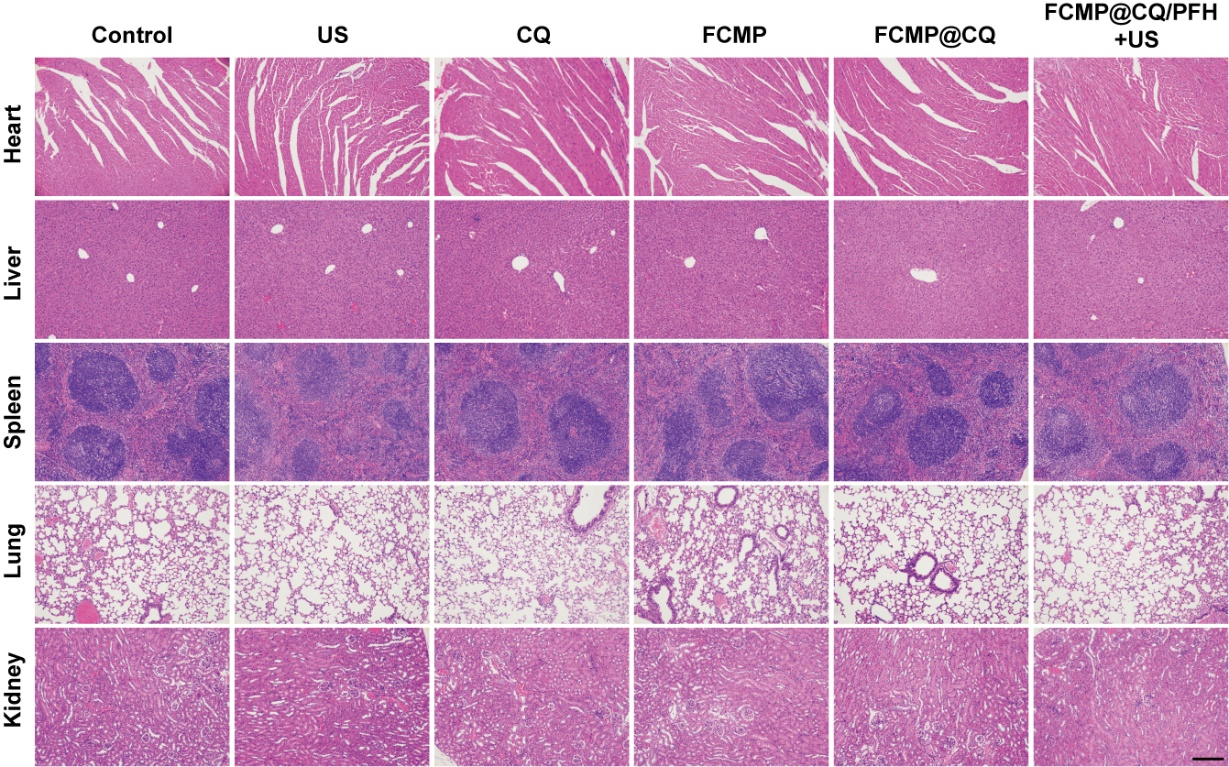


**Figure S29.** H&E staining in major organs of mice receiving different treatments. Scale bar: 100 μm.


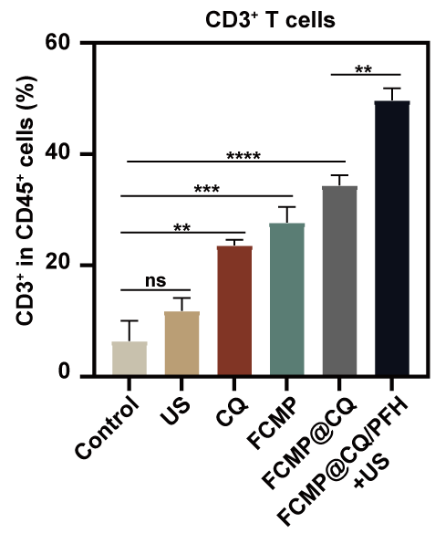


**Figure S30.** Percentages of CD3^+^ T cells gating on CD45^+^cells in subcutaneous MC38 tumor tissue (*n* = 4; mean ± SD). Statistical significance was determined by one-way ANOVA with Tukey’s test. **p* < 0.05, ***p* < 0.01, ****p* < 0.001, *****p* < 0.0001 and ns, no significant difference.


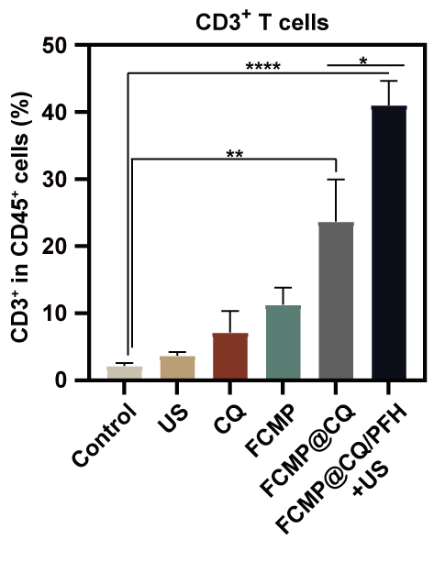


**Figure S31.** Percentages of CD3^+^ T cells gating on CD45^+^cells in 4T1 primary tumor tissue (*n* = 4; mean ± SD). Statistical significance was determined by one-way ANOVA with Tukey’s test. **p* < 0.05, ***p* < 0.01, ****p* < 0.001, *****p* < 0.0001 and ns, no significant difference.


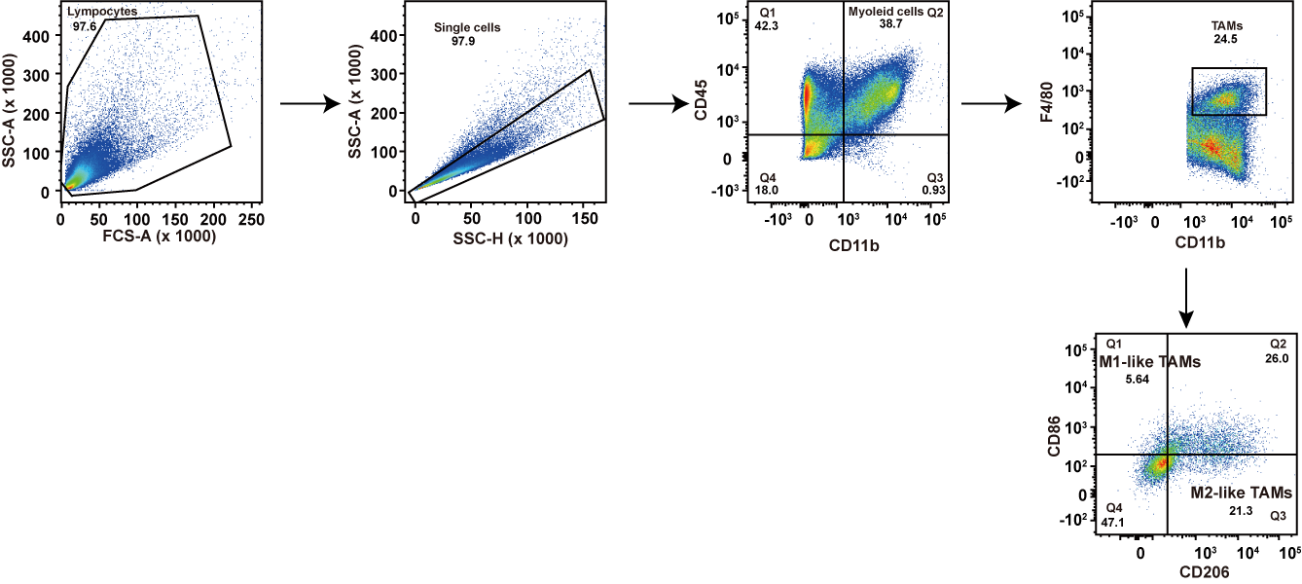


**Figure S32.** Gating strategy for identifying M1- and M2-like TAMs in tumor issue in Figure7B, C and Figure 8H, I.


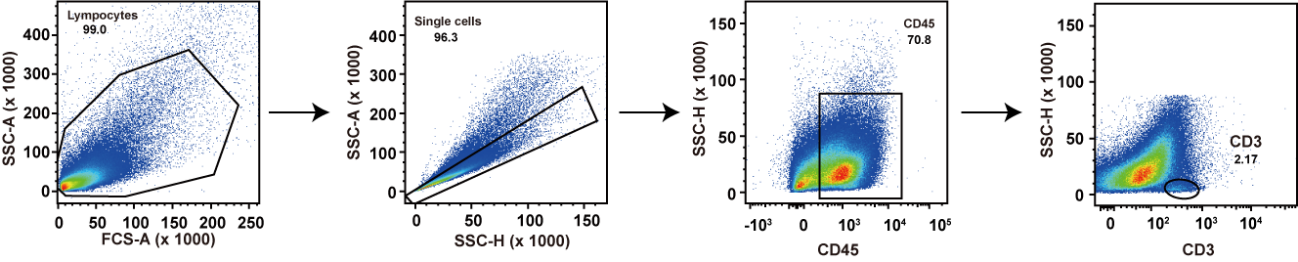


**Figure S33.** Gating strategy for CD3^+^ T cells analysis in tumor issue in in Figures S30 and S31.


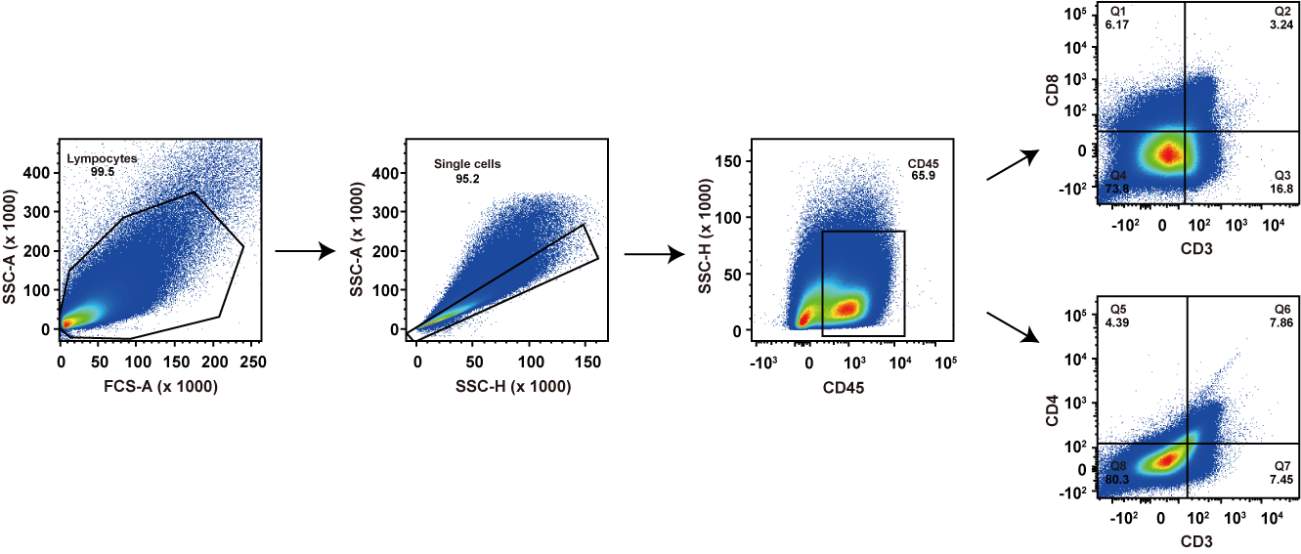


**Figure S34.** Gating strategy for CD8^+^ and CD4^+^ T cells analysis in tumor issue in Figure 7D, E and Figure 8J, K.


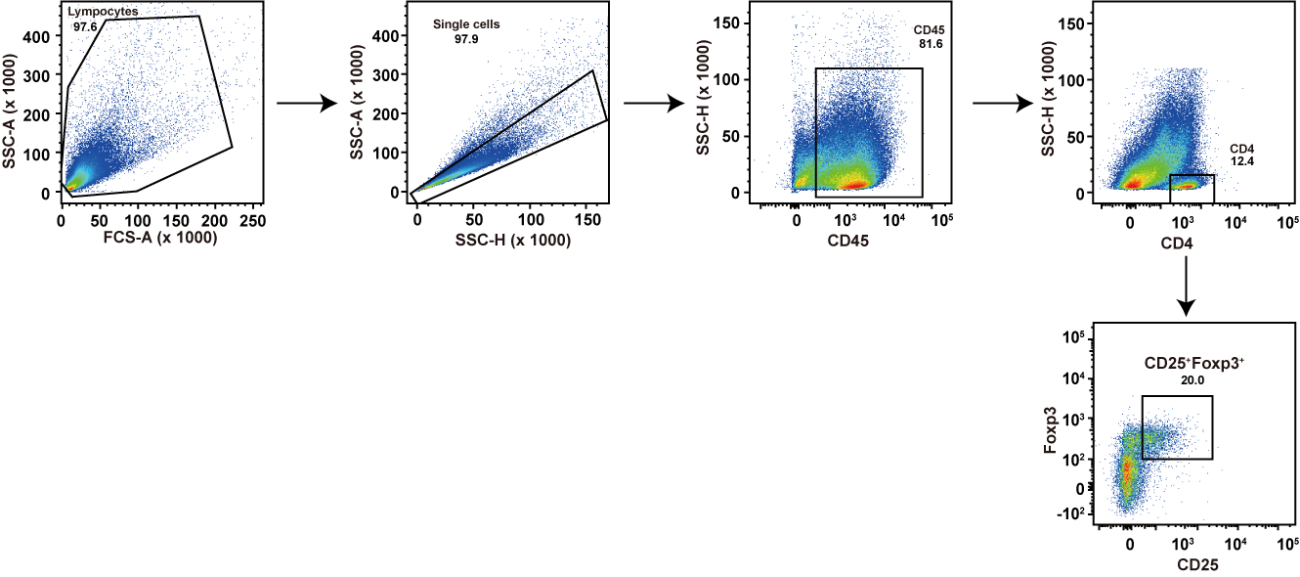


**Figure S35.** Gating strategy for Treg cells analysis in tumor issue in Figure 7F and Figure 8L.

**References**

[1] Y. Liang, L. Zhang, C. Peng, S. Zhang, S. Chen, X. Qian, W. Luo, Q. Dan, Y. Ren, Y. Li, B. Zhao, *Acta Pharm. Sin. B* **2021**, *11*, 3231.
